# fMRI reveals brainstem and cortical networks contribute to the instruction-dependent modulation of stretch-evoked motor responses

**DOI:** 10.1162/IMAG.a.1372

**Published:** 2026-09-18

**Authors:** Rebecca C. Nikonowicz, Neha A. Reddy, Michelle C. Medina, Molly G. Bright, Fabrizio Sergi

**Affiliations:** Department of Biomedical Engineering, UD College of Engineering, University of Delaware, Newark, DE, United States; Department of Physical Therapy and Human Movement Sciences, Feinberg School of Medicine, Northwestern University, Chicago, IL, United States; Department of Biomedical Engineering, McCormick School of Engineering and Applied Sciences, Northwestern University, Evanston, IL, United States; Department of Mechanical Engineering, UD College of Engineering, University of Delaware, Newark, DE, United States

**Keywords:** reticulospinal tract, instruction-dependent, fMRI, stretch reflexes

## Abstract

Reliable noninvasive measurement of human brainstem activity during motor control remains challenging due to small anatomical structures and physiological noise, yet it is essential for understanding descending contributions to rapid feedback control. We used brainstem-optimized whole-brain functional magnetic resonance imaging (fMRI) to examine whether task-dependent modulation of stretch-evoked motor responses in humans is associated with changes in activation within reticulospinal regions of the human brainstem. A behavioral validation experiment (N = 10), in which participants were instructed to resist or yield to brief wrist perturbations, confirmed task-dependent modulation of long-latency responses (LLRs). In a separate imaging cohort (N = 26), participants performed the same tasks during fMRI using an MRI-compatible robotic device and a multi-echo acquisition with physiological noise compensation. Imaging analyses revealed greater blood-oxygen-level-dependent signal during Resist than during Yield while controlling for background contraction and proprioceptive input, with activation distributed bilaterally across the pons and medulla in regions consistent with major reticulospinal nuclei. Laterality analyses demonstrated a rostrocaudal gradient, with relatively ipsilateral-biased activation in the medulla shifting toward more contralateral patterns in the pons. These findings indicate that instruction-dependent modulation of stretch-evoked motor responses is associated with measurable changes in human brainstem activation, providing evidence that reticulospinal regions contribute to task-dependent feedback control.

## 1 Introduction

The reticulospinal tract (RST) is a descending motor pathway that plays a critical role in postural control, gross limb movements, and motor recovery following neurological injury. Unlike the corticospinal tract (CST), which enables fine, distal control, the RST contributes to broader, bilateral muscle activation and appears to compensate for corticospinal damage in individuals with stroke or spinal cord injury (Baker, 2011; McPherson et al., 2018). Despite its functional significance, the RST remains poorly characterized in humans, largely due to its deep location in the brainstem and the absence of reliable, non-invasive techniques to quantify its activity. As a result, direct evidence of RST engagement during voluntary or task-modulated behavior in humans has been elusive.

One promising strategy to probe reticulospinal activity involves the study of long-latency responses (LLRs), stereotypical muscle responses occurring 50–100 ms after a muscle-stretching perturbation and likely involving both spinal and supraspinal processing (Kurtzer, 2014; Pruszynski et al., 2011). LLRs are modulated by task goals and instructions. Specifically, LLR amplitude (LLRa) will be higher if a participant attempts to resist a perturbation as opposed to yield to it (Lewis et al., 2006). This task-dependent gain scaling likely reflects supraspinal engagement and in fact, many studies have concluded that LLRs are generated and/or modulated by the primary motor cortex (M1), sensorimotor cortex (S1), premotor cortex, somatosensory areas, and secondary somatosensory cortex (Capaday et al., 1991; Kourtis et al., 2008; Marsden et al., 1977; Palmer & Ashby, 1992; Soteropoulos & Baker, 2020; Spieser et al., 2013).

While these studies emphasize the cortical contributions to LLRs, Shemmel et al. (2009) showed that transcranial magnetic stimulation (TMS) suppression of M1 does not diminish LLRa during a Resist condition, suggesting the goal-dependent component of LLRs is modulated elsewhere in the brain. One plausible candidate for processing such goal-dependent component of the LLRs is the reticular formation (RF), which originates the RST. Recordings from the pontomedullary RF in non-human primates have shown neuronal responses to both active finger flexion and extension and passive muscle stretch that precede onset of LLR EMG activity, providing evidence that some components of the LLR may be mediated through a trans-reticular pathway (Soteropoulos et al., 2012). The RF has also been strongly implicated in the startle response via the “StartReact” paradigm in which a startling acoustic stimulus triggers a faster than normal preplanned motor action (Carlsen et al., 2011; Valls-Solé et al., 1999). These StartReact responses occur at latencies under 70 ms, faster than typical voluntary reaction times, and are thought to rely on subcortical circuits rather than cortical drive alone (Carlsen et al., 2011; Valls-Solé et al., 1999). Supporting this theory, individuals with stroke or degenerated CST have delayed voluntary reactions but normal onset of StartReact (Honeycutt & Perreault, 2012). However, the relationship between startle responses and LLRs remains debated. Although Ravichandran et al. (2013) demonstrated a similar pattern of activity in the LLR epoch for participants attempting to quickly initiate an elbow movement with (1) a startling acoustic stimulus and (2) a perturbation, suggesting common underlying pathways, Forgaard et al. (2016, 2018) have demonstrated that mechanical perturbations can elicit rapid triggered responses in the absence of a measurable startle response. Thus, while overlap between StartReact and LLR-related responses suggests that subcortical pathways including the RST may contribute to rapid task-dependent motor responses, mechanical perturbation-evoked LLR modulation cannot be assumed to represent a startle-mediated response. Taken together, these findings implicate the RF, and by extension the RST, as a plausible contributor to task-dependent LLR modulation in humans, offering a non-invasive behavioral probe of RST function.

Despite growing evidence implicating the RST in rapid, goal-directed motor responses, most human studies have inferred its involvement indirectly through behavioral paradigms such as StartReact or through peripheral physiological measures including electromyography (EMG) amplitude and response latency. To the authors’ knowledge, no study has measured human brainstem activation associated with task-dependent modulation of LLRs during wrist perturbations. This gap reflects both anatomical and technical challenges: the RF lies deep within the brainstem and consists of small, densely packed nuclei, making it difficult to isolate function using noninvasive neuroimaging due to limited spatial resolution and substantial physiological noise in this region.

Studies using mechanical perturbations during fMRI are relatively rare because of the technical challenges associated with delivering such stimuli within the MR environment. One notable exception includes the work of Suminski et al. (2007), who combined an MR-compatible robotic wrist perturbation device with fMRI to investigate the neural correlates of wrist posture stabilization. Their study demonstrated distributed cortical and subcortical activation associated with online feedback control but did not examine brainstem activation. Consequently, the neural correlates of reflex-mediated control within the human brainstem remain largely unexplored.

To overcome these challenges, our group has developed StretchfMRI, a novel paradigm combining robotic perturbations, EMG, and functional magnetic resonance imaging (fMRI) to elicit LLRs and record muscle (EMG) and neural (fMRI) activity simultaneously (Zonnino et al., 2019). Preliminary studies have shown that neural activity associated with LLRs in the wrist flexors and extensors can be reliably measured in the RF (Nikonowicz & Sergi, 2024; Zonnino et al., 2021). Our approach leverages the Dual Motor StretchWrist (DMSW), an MRI-compatible robotic device that delivers controlled mechanical perturbations to the wrist, alongside brainstem-optimized whole-brain fMRI acquisition and analysis. By delivering rapid perturbations paired with “Yield” and “Resist” task instructions, we use LLR modulation as a behavioral probe to differentially engage descending motor pathways. Combined with region-specific hemodynamic response modeling, denoising tailored to the brainstem, and a multi-echo whole-brain fMRI sequence optimized to increase signal-to-noise in the brainstem (Reddy, Clements, et al., 2024), this method enables the visualization of neural activation in the RF. Using this experimental setup, we conducted a study to test two hypotheses: (H1) stretch-evoked fMRI signal in the RF is larger when participants are instructed to “resist” a perturbation compared with “yield” and (H2) brainstem activations show a rostrocaudal lateralization gradient that is consistent with the double reciprocal model of brainstem motor control (Davidson & Buford, 2006; Davidson et al., 2004; Sakai et al., 2009). Derived primarily from animal studies, this model proposes that the ipsilateral reticular formation preferentially facilitates flexor activation, while the contralateral reticular formation preferentially facilitates extensor activation. Along the pons-to-medulla axis, the model further predicts a lateralization gradient with flexor activation becoming increasingly ipsilateral in medullary regions. Although the framework provides a useful basis for interpreting brainstem motor organization, the extent to which these patterns generalize to human brainstem activation remains unclear. In addition to these primary hypotheses, we report behavioral and physiological validation outcomes, including EMG measures of stretch reflex modulation and whole-brain fMRI results to contextualize the brainstem findings within broader motor network activity. As such, this study aims to provide the first direct evidence of brainstem activation modulated by fast-feedback motor task demands in humans, offering a new, non-invasive approach to studying reticulospinal contributions to motor control.

## 2 Materials and Methods

### 2.1 Participants

In total, 10 healthy individuals (age: 25±4.45 years , 7M/3F) and 26 healthy individuals (age: 24±3.41 years , 12M/14F) participated in Experiment 1 (conducted in a mock MRI scanner) and Experiment 2 (conducted in an MRI scanner), respectively. All participants self-reported as right handed and free from neurological disorders and orthopedic/pain conditions affecting the right wrist. Written informed consent was obtained from all participants for being included in the study. The study was approved by the investigation review board of the University of Delaware under protocol no. 1097082-11 and conducted in accordance with the Declaration of Helsinki.

### 2.2 Dual Motor StretchWrist

**Fig. 1.**
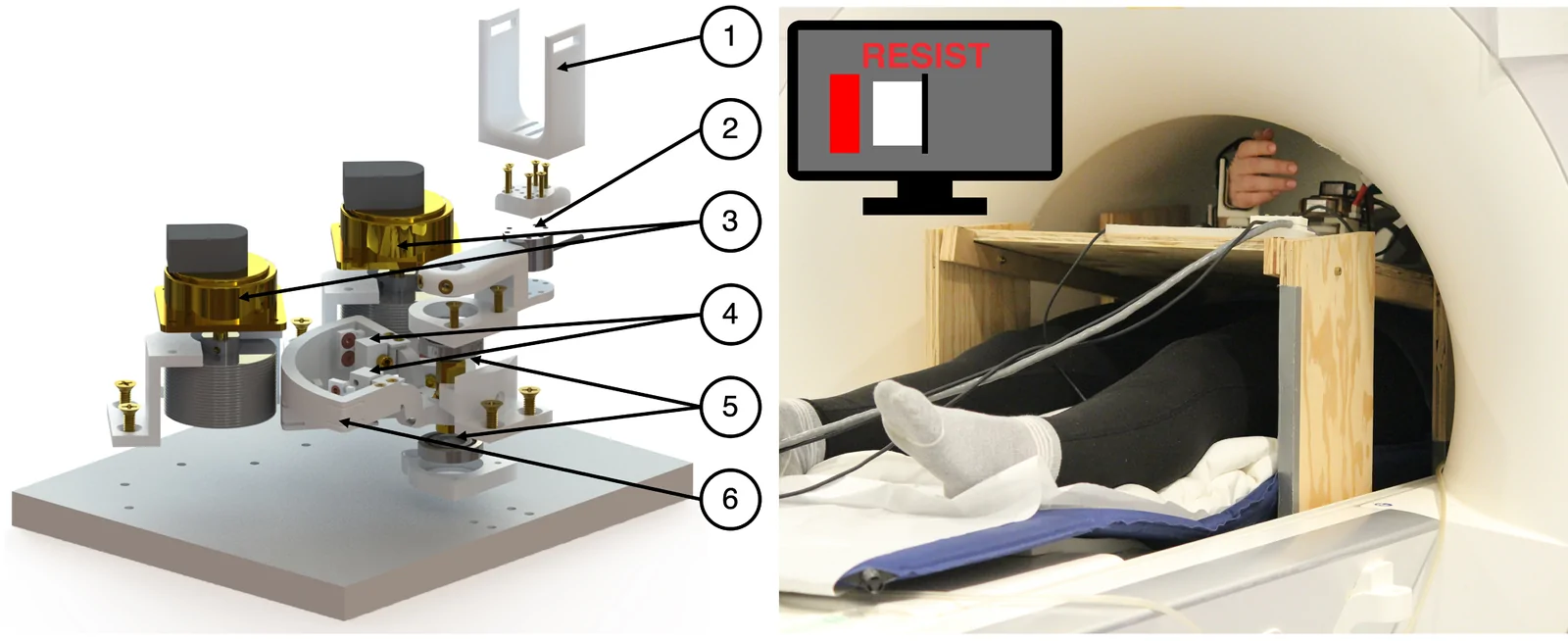
(Left) Exploded view of the modeled Dual Motor StretchWrist with labels for (1) hand support, (2) MR-compatible force–torque sensor, (3) MR-compatible piezoelectric ultrasonic motors, (4) tensioning mechanisms, (5) ceramic support bearings, and (6) capstan arc. (Right) A participant and the DMSW inside of the MRI scanner and a sample view of the Resist cued task.

A one degree-of-freedom robot, the Dual Motor StretchWrist (DMSW), was used to deliver precise extension perturbations to the right wrist to condition LLRs in the wrist flexors. The DMSW features two ultrasonic piezoelectric motors (EN60 Motor Shinsei Motor Inc., Japan) connected in parallel and has a maximum torque of 6 Nm, range-of-motion of 110 degrees, and peak, unloaded velocity of 900 deg/s. The capstan transmission allows for motion transfer from the motors to the end effector and the entire device is manufactured with MR-compatible materials including 3D printed plastic (RS- F2-FPWH-04, Formlabs, Inc., MA, USA), bronze, and brass fasteners and structural supports, ceramic bearings (Boca Bearings, Boynton Beach, FL, USA), and a six axis, MR-compatible force–torque sensor (Mini27Ti, ATI Industrial Automation, Apex, NC). The device and its control are described in detail in Nikonowicz and Sergi (2024) (Fig. 1).

### 2.3 EMG in the mock MRI scanner

Electromyography (EMG) data were collected in Experiment 1 using only surface electrodes (Delsys Trigno Duo, Natick, MA) at a sampling rate of 1926 Hz. EMG signals were recorded from the flexor carpi radialis (FCR) and extensor carpi ulnaris (ECU).

### 2.4 Yield–resist task

**Fig. 2.**
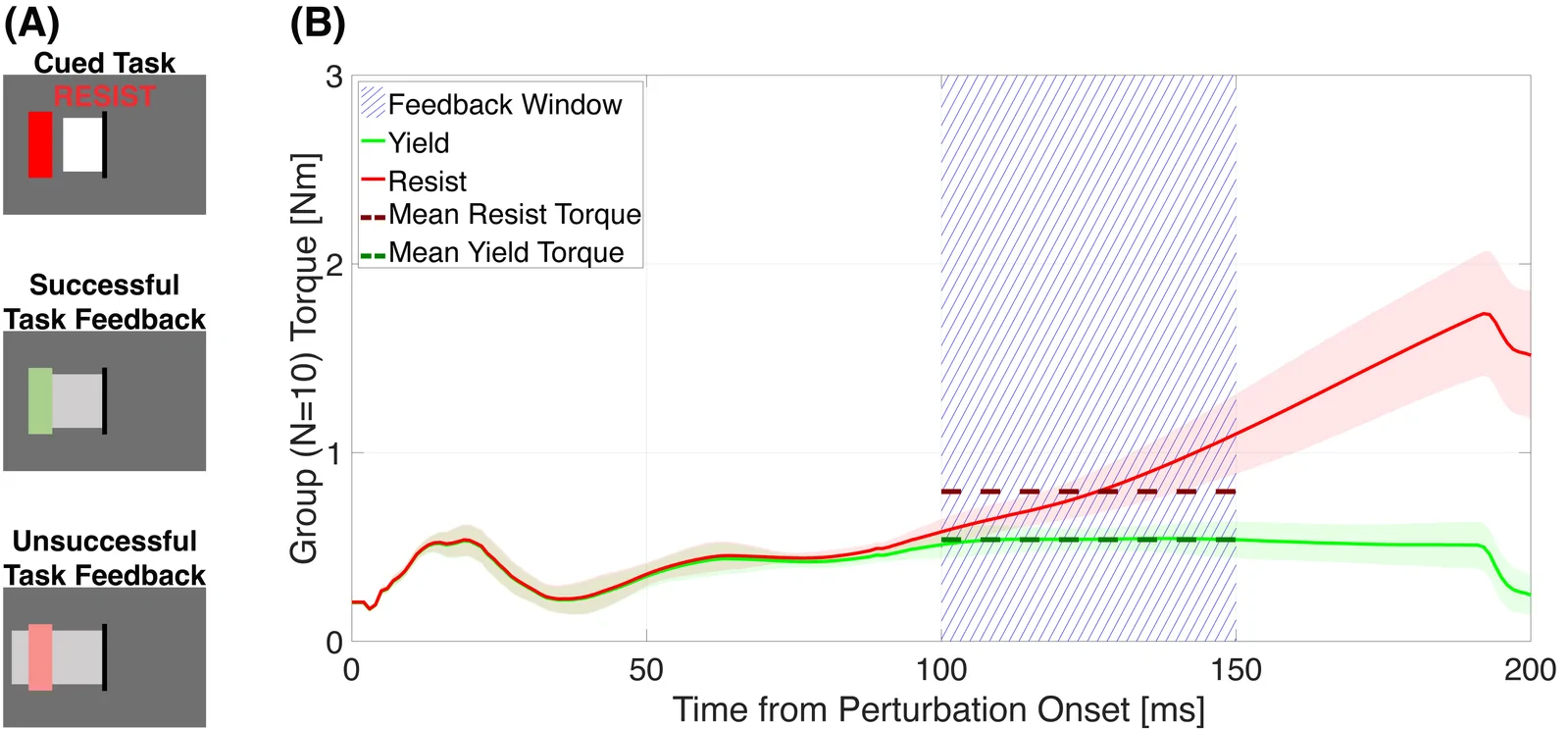
(A) Sample participant views during the task. Cued Task: what the participant sees when the task is cued, before the perturbation. The red box is the target, the white box is their live measured flexion torque, the black line is 0 Nm, and the task instruction is at the top. Successful Task Feedback: what the participant sees after a perturbation when they applied the desired level of flexion torque during the perturbation where the green box is the successfully met target and the gray box is the average torque measured in the [100 150] ms post-perturbation onset. Unsuccessful Task Feedback: what the participant sees after a perturbation when they applied too much torque during the perturbation where the pink box is the unmet target. (B) Average of the torque measured by the DMSW during perturbations in Experiment 1, averaged across perturbations and participants. Thick lines represent the average and shaded areas represent ± 1 s.d., indicating within-group variance. The area where torque was averaged and then displayed to participants is highlighted by blue hatch marks.

During task sessions, participants were exposed to a blocked design of perturbations to the right wrist. Before each perturbation, participants were cued via a visual interface to produce a flexion torque of 0.2±0.035 Nm (Fig. 2A). After holding this torque for a duration randomly selected from a uniform distribution with extremes 400 and 800 ms, a perturbation was delivered in the wrist extension direction, resulting in stretch of the wrist flexor muscles. Perturbations were delivered at either 150 deg/s or 35 deg/s for a duration of 200 ms. After the perturbation, participants received visual feedback on their measured torque (details below) for 2 seconds before being returned to the start position at 35 deg/s. Once returned to start, the next cued flexion torque appeared immediately. Each perturbation trial lasted approximately 3.75 seconds.

**Task Instructions:** In both experiments, participants were exposed to three perturbation conditions: Yield, Resist, and Slow. During Yield and Resist conditions, participants were instructed to either “Yield” to the perturbation or “Resist” the perturbation, and perturbations were delivered at 150 deg/s. During Slow conditions, participants were instructed to “Yield” to the perturbation and perturbations were delivered at 35 deg/s. This Slow condition was included as it is known to minimally/not elicit stretch responses in wrist muscles (Nikonowicz & Sergi, 2024; Weinman et al., 2021).

**Task Length:** In Experiment 1, participants completed one task session. In Experiment 2, participants completed two task sessions. Each session comprised 5 blocks of each condition (15 total blocks). Each block consisted of 8 perturbations of the same condition lasting approximately 30 seconds, resulting in a total of 40 perturbations per condition, or a total of 120 total perturbations in each task session. Block order was randomized ([Y R R S Y Y R S Y R S S Y R S]) but held consistent across sessions and participants. In Experiment 2, each block was interleaved with 30 seconds rest periods. For each participant, the total number of volumes collected varied, but on average, each session lasted approximately 16 minutes. A Familiarization session preceded the task, during which participants experienced 40 perturbations per condition to become comfortable with the robotic interface and task instructions.

**Task Torque Feedback:** During the task sessions, visual feedback was provided after all perturbations to standardize resistance level in a subject-specific way. Visual feedback was provided based on the average torque value measured in the time comprised between 100 and 150 ms post-perturbation onset, relative to the values measured on the same individuals and perturbation conditions during Familiarization. Specifically, participants were asked to apply 100 ± 40% the torque measured in Familiarization Yield during Task Yield, 200 ± 40% the torque measured in Familiarization Yield during Task Resist, and 100 ± 40% the torque measured in Familiarization Slow during Task Slow. Trials were considered successful if the measured torque fell within the prescribed range. If the trial was successful, the goal torque target turned green. If the trial was unsuccessful, the goal torque target remained red.

During the Resist condition in Familiarization sessions, visual feedback was provided after each perturbation, based on the average torque measured in the time comprised between 100 and 150 ms post-perturbation onset. This time frame was chosen to include a 50 ms delay from the [50 100] ms time interval typically considered for LLRs for upper extremity muscles. The delay was set to 50 ms to account for the electromechanical delay and to avoid participant success due to a “voluntary” response (typically considered as EMG signal measured >100 ms). Participants were instructed to apply 200 ± 40% the torque that was measured in the same time range during the Yield condition, delivered just prior to the Resist condition during Familiarization (Fig. 2).

At the beginning of each session, participants were asked to complete five 1 second isometric contractions in extension at 0.2±0.035 Nm for EMG normalization purposes.

### 2.5 MRI procedures

Imaging was performed using a Siemens 3T Prisma MRI system with a 64-channel head coil. Functional scans were acquired across two task sessions lasting approximately 15 minutes each. Scans were collected using a multiband multi-echo gradient-echo echo planar imaging sequence (TR = 2.2 seconds, TEs = 13.4/39.5/65.6 ms, FA = 90 degrees, MB factor = 2, GRAPPA = 2, voxel resolution = 1.731 x 1.731 x 4 mm^3^, 44 slices, FOV = 180 mm, matrix size 104 x 104). Voxel resolution was kept small in-plane and longer out-of-plane, with voxel alignment to the brainstem to account for the orientation of brainstem nuclei that are very small but oblong axially.

A structural T1-weighted multi-echo magnetization-prepared rapid acquisition with gradient echo (MPRAGE) scan (TR = 2.17 seconds, TEs = 1.69/3.55/5.41 ms, TI = 1.16 seconds, FA = 7 degrees, FOV = 256 x 256 mm^2^, and voxel resolution = 1 x 1 x 1 mm^3^) was acquired at the end of each fMRI experiment for registration and normalization to a common space. The three echo images were combined via root mean square.

### 2.6 Data processing and statistical analysis

#### 2.6.1 EMG data processing

A standard processing pipeline was used to process EMG data: band-pass filtering (4th order Butterworth 20–250 Hz) to remove motion artifacts and high frequency noise, signal rectification, and low-pass filtering (4th order Butterworth 60 Hz) to extract the envelope of the EMG signal.

FCR EMG signal was normalized by dividing the processed signal by the average signal measured in the last 200 ms during background contraction before perturbation onset across all perturbation trials. ECU EMG signal was normalized by dividing the processed signal by the average signal measured in five 1 second isometric contractions in extension conducted at the beginning of each session.

LLRa for each trial was calculated as the mean EMG signal 50–100 ms post-perturbation onset, as has been done in previous studies (Nikonowicz & Sergi, 2024; Weinman et al., 2021). Additionally, background amplitude (BKGDa) and short latency response amplitude (SLRa) were calculated as the mean EMG signal 200–0 ms pre-perturbation onset and 25–50 ms post-perturbation onset, respectively. SLRa was included as an outcome because it is understood to be a spinal reflex and thus should not be affected by supraspinal feedback incorporating task goals.

#### 2.6.2 Torque data processing

Torque data were processed using Matlab 2023a (Mathworks, Inc. Natick, MA, USA). For each perturbation trial, torque response amplitude was calculated as the mean torque measured 100–150 ms post-perturbation onset. Success rates for each instruction condition were defined as whether the trial was in the red or green condition as displayed by the visual feedback (Fig. 2).

#### 2.6.3 Statistical analysis

EMG and torque data were analyzed at a group level using a linear mixed model with condition (Yield, Resist, Slow) as the main effect and participant and the interaction of participant and condition as random effects. For participant-level analysis, no random effects were included, and a one-way ANOVA was used instead. Post-hoc Tukey tests were used to test for significant differences between conditions. For post-hoc tests, the Cohen’s effect size for paired samples *d_z_* was computed as the difference of the means divided by the standard deviation of the difference. All statistical analyses were conducted using JMP 17 Pro (SAS Institute Inc., Cary, NC, USA).

#### 2.6.4 fMRI data processing

Analysis of fMRI data was performed using a custom pipeline that integrates functions from FSL (version 6.0.7.7) (Jenkinson et al., 2012), AFNI (version 24.3.06) (Cox, 1996), and SPM12 (Wellcome Department of Cognitive Neurology, London, UK) running on Matlab 2023a. Structural T1-weighted images for each participant were processed using FSL using bias field correction and brain extraction. Functional data were preprocessed using FSL and AFNI. Head motion realignment was estimated for the first echo data referencing the single band reference image taken at the beginning of the scan and then applied to all echo timeseries. All images were brain extracted and distortion corrected before Tedana (version 24.0.2) was used to calculate a ${\text{T}}_{2}^{\text{*}}$-weighted combination of the three echo datasets, producing an optimally combined timeseries. Multi-echo independent component analysis (ME-ICA) was performed on the optimally combined dataset using Tedana and resulting components were manually classified as accepted (signal of interest) or rejected (noise) (Reddy, Zvolanek, et al., 2024). A 4 mm spatial smoothing was applied to functional data for whole-brain analyses, while smoothing was left out of the preprocessing pipeline for brainstem-specific analyses.

SPM12 was used for first- (participant-level) and second-level (group-level) analyses. Functional datasets were treated as blocked design datasets rather than event design datasets to avoid dependence on accurate transient hemodynamic response modeling as brainstem hemodynamics are different than that of cortical hemodynamics. Separate first-level analyses were conducted to determine whole-brain and brainstem-specific activation. The same general linear model was used for both analyses but the model regressors were convolved with a standard hemodynamic response function for whole-brain analysis and a brainstem-specific hemodynamic response function for brainstem-specific analysis (Kim et al., 2022; Zonnino et al., 2021). The brainstem-specific hemodynamic response function peaks earlier and has a smaller, earlier undershoot than the standard hemodynamic response function, reflecting the faster and more transient blood-oxygen-level-dependent response observed in brainstem nuclei compared with cortical regions. The neural response was modeled as

$$y=\beta_{R}H_{R}+\beta_{Y}H_{Y}+\beta_{S}H_{S}+\beta_{0}+\beta_{NUI}R_{NUI},$$ (1)

where *H_R_*, *H_Y_*, and *H_S_* are the task regressors (rectangular functions convolved with the hemodynamic response function) and *R_NUI_* is a set of 24+ nuisance regressors. Nuisance regressors included 18 physiological noise regressors (6 cardiac, 8 respiratory, 4 interaction) and 6 head movement regressors calculated using the RETROICOR algorithm via the TAPAS PhysIO Toolbox (Kasper et al., 2017). Additionally, nuisance regressors included rejected regressors identified via multi-echo independent component analysis (ME-ICA) created using Tedana, as was done in Reddy, Zvolanek, et al. (2024) and orthogonalized to all other regressors plus the accepted components from Tedana’s ICA, via AFNI’s 3dTproject (Medina et al., 2025). Note that unsuccessful trials were not accounted for by additional regressors.

General linear model estimation was used to identify voxels whose signal was significantly modulated by any experimental condition relative to rest (Slow > 0: β*_S_* > 0, Yield > 0: β*_Y_* > 0, Resist > 0: β*_R_* > 0), or significantly more in any experimental condition relative to the others (Resist > Yield: $\beta_{R}-\beta_{Y}>0$ , Resist > Slow: $\beta_{R}-\beta_{S}>0$ , Yield > Slow: $\beta_{Y}-\beta_{S}>0$ ). Then, participant-specific statistical parametric maps were used as inputs for second-level group analysis. For brainstem-specific analysis, threshold-free cluster enhancement (TFCE) (Smith & Nichols, 2009) was performed within a mask of the brainstem created by thresholding the brainstem region of the Harvard-Oxford Subcortical Atlas (Desikan et al., 2006; Frazier et al., 2005; Goldstein et al., 2007; Makris et al., 2006) at 50%. Group-level analysis maps are reported using family-wise error correction (α<0.05 ) and significance is set to p<0.05 . A more conventional, supplementary brainstem-specific analysis (Supplementary Figs. S15–S27 available in Supplementary Materials) was conducted without ME-ICA rejected regressors and with a small-volume correction to control for the family-wise error rate (α<0.05 ) in the same region of interest (Poldrack et al., 2008).

To break down the measured activation into clusters, SPM12’s *spm_clusters* was used on the resulting TFCE-corrected maps and clusters including more than five voxels were reported. Within these clusters, peaks of activation were identified automatically. Clusters were further broken down by anatomical region (Midbrain, Pons, and Medulla, defined based on the FreeSurfer Brainstem Substructures Atlas (Iglesias et al., 2015), thresholded at 50%), and by laterality (left or right). Within each anatomically divided cluster, the three peaks with the greatest statistical parameters are reported for concise summary of cluster maxima, separately for each side.

To identify which nuclei may have contributed to the measured cluster activation, we compared the cluster maps to nuclei atlases from Brainstem Navigator (Bianciardi et al., 2015, 2018; García-Gomar et al., 2019, 2022; Singh et al., 2020, 2021). For each cluster, we quantified spatial overlap with atlas-defined nuclei. A nucleus was considered a potential contributor if the overlapping voxels accounted for ≥5% of the cluster volume. This threshold was used as a heuristic to identify nuclei with meaningful spatial overlap while avoiding attribution based on very small intersections between atlas labels and clusters. Qualitatively similar candidate nuclei were identified when modestly varying this threshold. Given the small size of many brainstem nuclei and the spatial resolution of our fMRI sequence, the atlas-based overlap analysis should be interpreted as identifying nuclei that are spatially consistent with the observed clusters rather than definitively localizing activation to specific structures.

We computed three outcomes to quantify laterality of the group-level activations in the brainstem at each axial slice (*z *= const). These metrics were designed to capture both the spatial distribution and magnitude across hemispheres and were conceptually derived from laterality index approaches commonly used in neuroimaging to quantify dominance of functional activation (Seghier, 2008). In all these calculations, we used data from all voxels in the ROI (defined as the union of Midbrain, Pons, Medulla from the FreeSurfer Atlas), with non-negative t-scores resulting from each of the three contrasts described above (R > 0, R > Y, R > S). First, we computed the sum of *t* scores (*t_sum_*) for the left (*l*) and right (*r*) sides as an indicator of intensity and extent of activation from either side, as

$$t_{sum,l}=\overset{n_{l}}{\underset{i=1}{\sum \;}}t_{i}$$ (2)

$$t_{sum,r}\;=\sum_{j=1}^{n_{r}}\;t_{j},$$ (3)

where *i* and *j* are indices corresponding to each voxel with non-negative score in a given slice, within the ROI, and *n_l_* and *n_r_* are the number of voxels with non-negative activation on the left and right sides, respectively. Our second outcome was the *t* score moment (*M*), which scales the contribution of each supra-threshold voxel proportionally to the lateral distance of each voxel from the midline (*x*), as

$$M_{l}=\overset{n_{l}}{\underset{i=1}{\sum \;}}t_{i}\cdot x_{i}$$ (4)

$$M_{r}\;=\sum_{j=1}^{n_{r}}\;t_{j}\cdot x_{j}.$$ (5)

We then combined the outcomes above into the third outcome, which would indicate the lateral coordinate of the single voxel that would produce the same amount of moment imbalance for a given slice as the entire distribution of *t* scores in that slice. In analogy with the definition of center of mass in mechanical systems, we defined this outcome as center of activation (COA), with the same units as *x* (thus mm), which would be positive to indicate right dominance of the spatial distribution of t-scores, and negative otherwise.

$$COA=\frac{M_{r}-M_{l}}{t_{sum,l}+t_{sum,r}}.$$ (6)

For whole-brain analysis, locations of significant activation were determined using FSL’s Juelich Histological Atlas, Harvard-Oxford Cortical Structural and Subcortical Atlases, and SUIT Probabilistic Cerebellum Atlas (Amunts et al., 2020; Desikan et al., 2006; Diedrichsen et al., 2009; Frazier et al., 2005; Goldstein et al., 2007; Makris et al., 2006).

## 3 Results

### 3.1 Protocol validation in the mock scanner

#### 3.1.1 Muscle activity and torque performance during perturbations

**Fig. 3.**
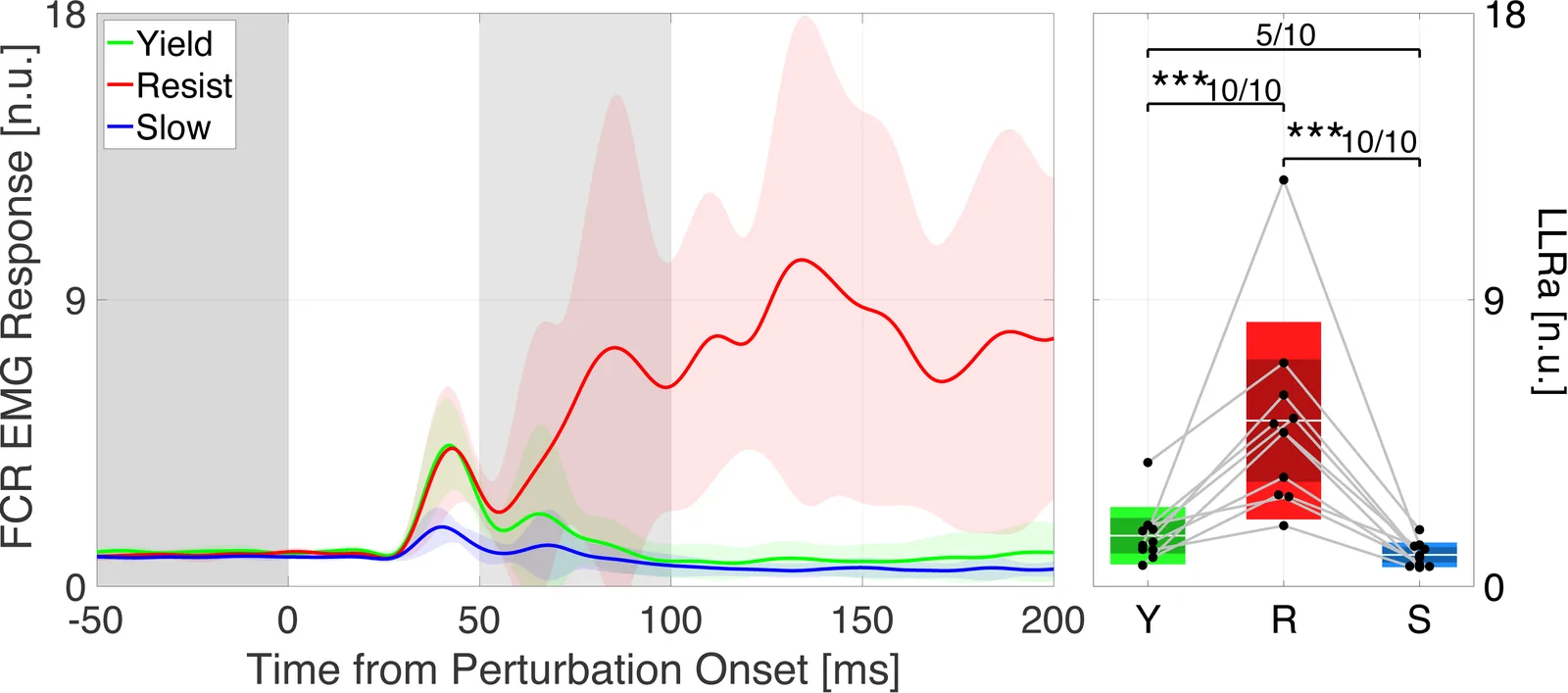
(Left) Group average of the FCR EMG measured in each perturbation condition across both task sessions in Experiment 1. The darker gray-shaded region indicates time before perturbation onset and includes part of the background EMG activity. The lighter gray-shaded region indicates the region of expected LLR activity. Thick lines represent the average and shaded areas represent ± 1 standard deviation (s.d.), indicating within-group variance. (Right) Box plots showing the distribution of LLRa across participants with mean (horizontal white line), mean ± 1 standard error of the mean (dark shaded area), and mean ± 1 s.d. (light shaded area). Black dots indicate LLR mean of individual participants. Number of asterisks indicates significance level (*p<0.05 , **p<0.005 , ***p<0.001 ).

Group-level statistical analysis of FCR EMG data collected in Experiment 1 shows significant effects of instruction for all the outcomes considered (i.e., FCR activation during background, SLRa, and LLRa). LLRa in FCR (primary outcome) was greater for Resist than for both Yield (Δ*LLRa_R_*_−_*_Y_*: 3.6141 ± 1.9026, p<0.001 , *d_z_* = 1.1773) and Slow (Δ*LLRa_R_*_−_*_S_*: 4.2140 ± 1.7979, p<0.001 , *d_z_* = 1.4527) conditions. Activity during Yield was not greater than Slow (Δ*LLRa_Y_*_−_*_S_*: 0.5999 ± 0.3788, p=0.7257 , *d_z_* = 0.9815), but on an individual participant level, LLRa was greater for Yield than for Slow for 5 of 10 participants (Fig. 3). Background activity was greater for Yield than for both Resist (Δ*BKGDa_Y_*_−_*_R_*: 0.1103 ± 0.1126, p=0.0063 , *d_z_* = 0.6069) and Slow (Δ*BKGDa_Y_*_−_*_S_*: 0.1522 ± 0.0722, p<0.001 , *d_z_* = 1.3066) conditions, but activity during Resist was not significantly different than during Slow (Δ*BKGDa_R_*_−_*_S_*: 0.0419 ± 0.0987, p=0.448 , *d_z_* = 0.2633). Importantly, the largest LLR responses occurred in the Resist condition despite similar background activation between Resist and Slow, suggesting that the observed LLR modulation reflects task-dependent feedback control rather than differences in baseline muscle activation.

For the SLR time window, FCR activity was greater for Resist (Δ*SLRa_R_*_−_*_S_*: 1.3794 ± 0.5440, p<0.001 , *d_z_* = 1.5717) and Yield (Δ*SLRa_Y_*_−_*_S_*: 1.4320 ± 0.4271, p<0.001 , *d_z_* = 2.0780) than for Slow, but not significantly different between Yield and Resist (Δ*SLRa_Y_*_−_*_R_*: 0.0526 ± 0.3176, p=0.9702 , *d_z_* = 0.1026). Detailed fixed effects results are available in Table 1.

**Table 1.** Mixed model results: Fixed effects for all validation experiment outcomes.

FCR RFXa	N par	DF Den	F Ratio	Prob>F
Instruction	2	18.0	17.1209	**<0.0001**
FCR BKGDa				
Instruction	2	54.7	10.4247	**<0.0001**
FCR SLRa				
Instruction	2	18.0	26.2391	**<0.0001**
ECU BKGDa				
Instruction	2	18.0	7.0611	**0.0054**
Avg. FB Torque				
Instruction	2	18.0	136.2297	**<0.0001**

Bold values indicate statistical significance.

**Fig. 4.**
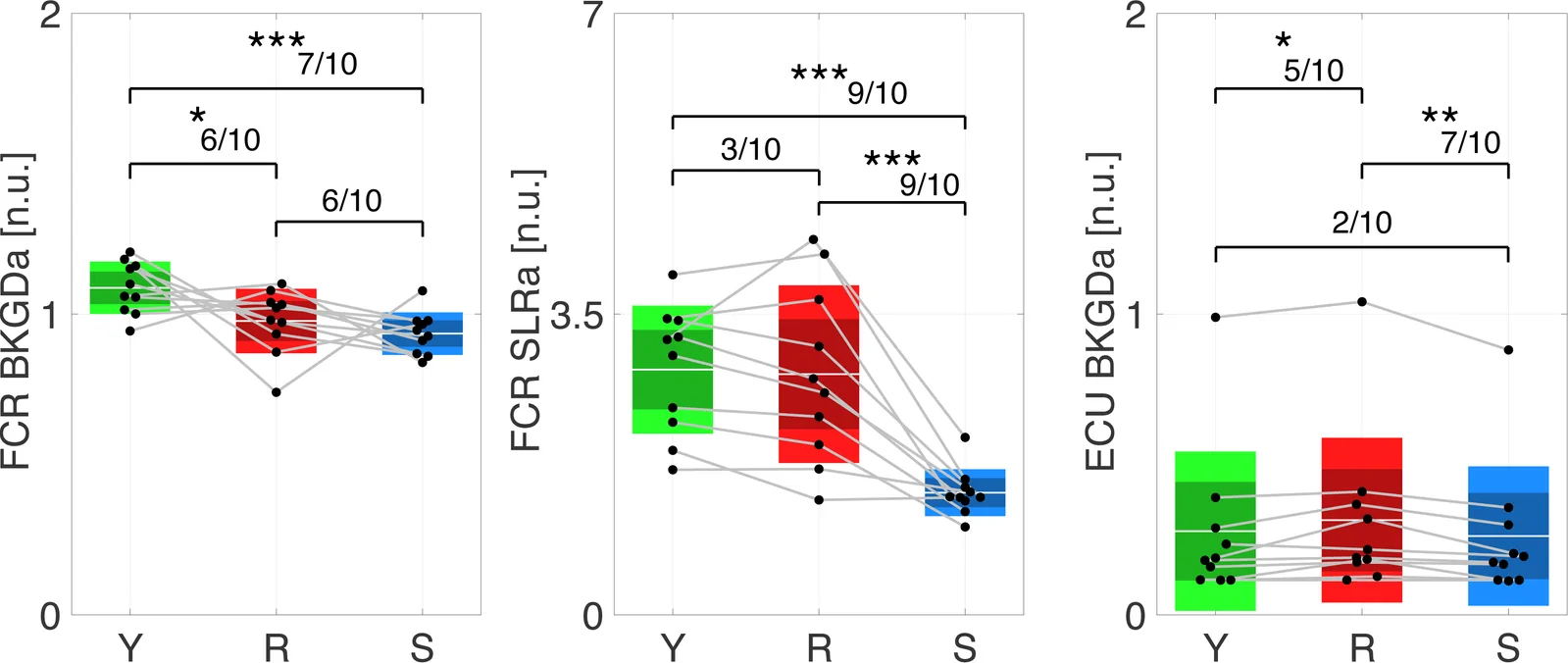
Secondary EMG outcomes from protocol validation. As in Figure 3 (right), all box plots show the distribution of average, normalized EMG across participants with mean (horizontal white line), mean ± 1 standard error of the mean (dark shaded area), mean ± 1 s.d. (light shaded area), and black dots indicate mean of individual participants. (Left) Background FCR EMG. (Center) Short latency response FCR EMG. (Right) Background ECU EMG. Number of asterisks indicates significance level (*p<0.05 , **p<0.005 , ***p<0.001 ).

Group-level statistical analysis of background ECU EMG data shows a significant effect of instruction. Specifically, activity during Resist was greater than during Slow (Δ*BKGDa_R_*_−_*_S_*: 0.0526 ± 0.0326, p=0.0048 , *d_z_* = 1.0008) and to Yield (Δ*BKGDa_R_*_−_*_Y_*: 0.0366 ± 0.0279, p=0.0499 , *d_z_* = 0.8135), but activity was not significantly different between Yield and Slow (Δ*BKGDa_Y_*_−_*_S_*: 0.0159 ± 0.0231, p=0.5199 , *d_z_* = 0.4279). This pattern is consistent with increased antagonist co-contraction during the Resist condition, which may reflect stabilization of the wrist in preparation to counteract the imposed perturbation (Fig. 4).

**Fig. 5.**
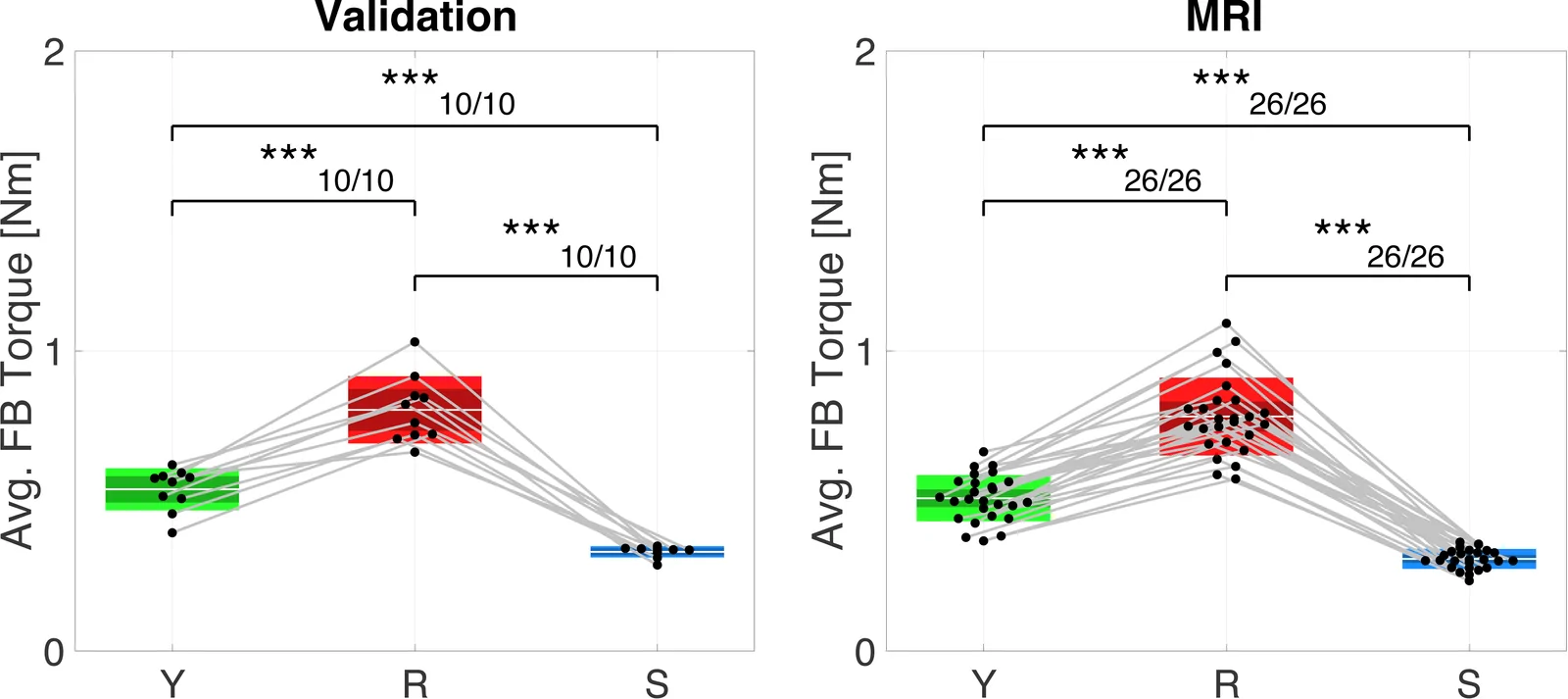
Average torque measured in the feedback window. (Left) Validation experiment, (right) MRI experiment. As in Figure 3 (right), box plots show the distribution of average feedback window torque across participants with mean (horizontal white line), mean ± 1 standard error of the mean (dark shaded area), and mean ± 1 s.d. (light shaded area). Black dots indicate mean of individual participants. Number of asterisks indicates significance level (*p<0.05 , **p<0.005 , ***p<0.001 ).

**Fig. 6.**
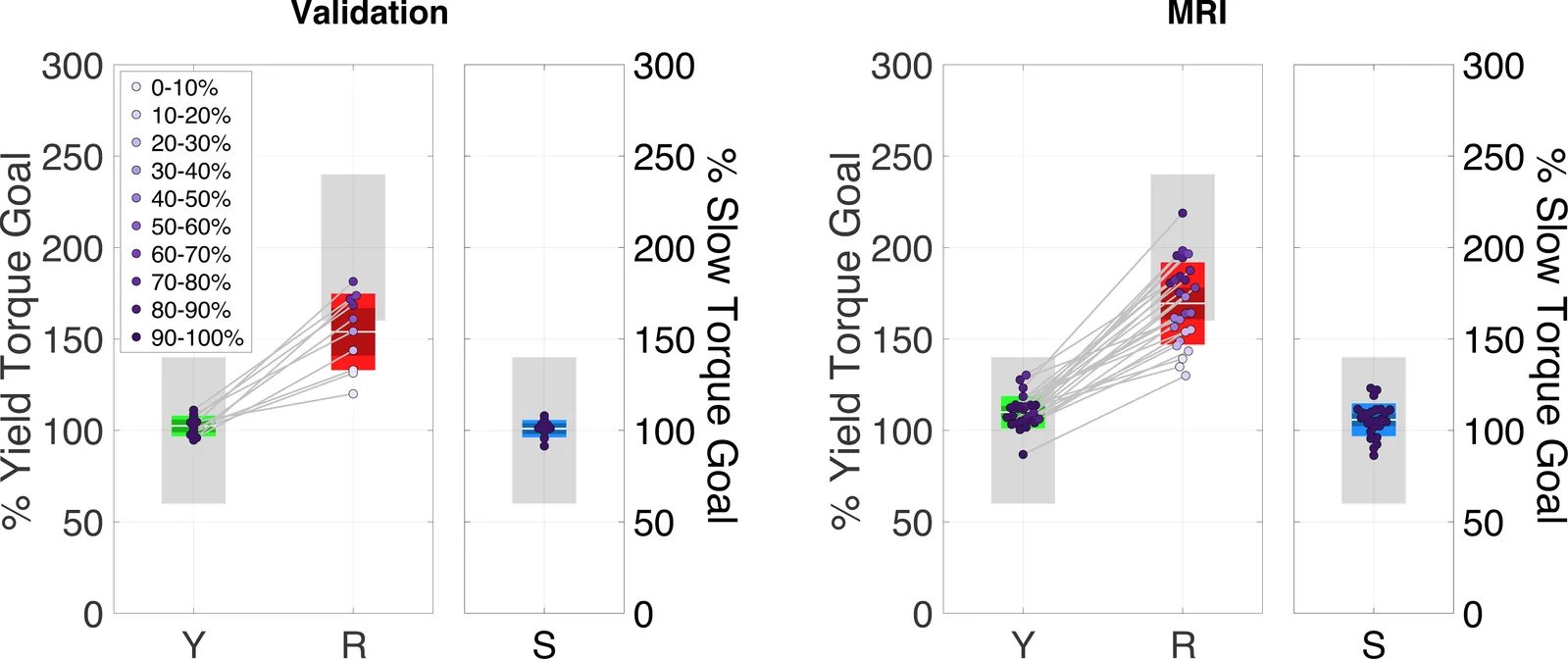
Average torque measured in the feedback window and normalized by torque goal with success rates for each individual participant. (Left) Validation experiment, (right) MRI experiment. As in Figure 3 (right), box plots show the distribution of average feedback window torque across participants with mean (horizontal white line), mean ± 1 standard error of the mean (dark shaded area), and mean ± 1 s.d. (light shaded area). Dots indicate mean of individual participants with the color indicating success rate.

Group-level statistical analysis of torque data shows a significant effect of instruction for torque measured in the feedback window (Fig. 5). Resist torque was larger than Yield (Δ*T_R_*_−_*_Y_*: 0.2646 ± 0.0615, p<0.001 , *d_z_* = 2.6662) and Slow (Δ*T_R_*_−_*_S_*: 0.4729 ± 0.0673, p<0.001 , *d_z_* = 4.3528) and Yield torque was larger than Slow (Δ*T_Y_*_−_*_S_*: 0.2083 ± 0.0344, p<0.001 , *d_z_* = 3.7493). These results were the same on an individual participant level for every participant. Significant effects on torque were measured across all conditions even though participants were not always able to modulate torque as cued by biofeedback. The average torque success rate varied between conditions, and ranged from almost perfect in the Yield and Slow conditions (Y: 99.63±0.92% , S: 100%) to lower levels of performance in the Resist condition (38.00±29.93% ) (Fig. 6, left). Although Yield and Slow conditions did not consistently differ in reflex EMG responses, torque differed across all conditions. This difference likely reflects variation in wrist motion and passive mechanical interaction between the hand and the imposed perturbation rather than differences in voluntary muscle activation.

Overall, these results indicate that task instruction selectively modulated LLRs, with greater LLRa during Resist than during Yield and Slow, while SLR amplitudes were similar between Resist and Yield. These findings are consistent with prior work showing that goal-dependent modulation primarily affects the long-latency component of stretch responses.

### 3.2 Perturbations in the MRI scanner

#### 3.2.1 Participant torque performance

In absence of in-scanner EMG data, torque was used as a proxy for task performance in the MRI experiment. Group-level statistical analysis of torque data shows a significant effect of instruction for torque measured in the feedback window, consistent with expectations. Consistent with the out-of-scanner validation experiment, Resist torque was larger than Yield (Δ*T_R_*_−_*_Y_*: 0.2723 ± 0.0397, p<0.001 , *d_z_* = 2.6392) and Slow (Δ*T_R_*_−_*_S_*: 0.4744 ± 0.0438, p<0.001 , *d_z_* = 4.1614) and Yield torque was larger than Slow (Δ*T_Y_*_−_*_S_*: 0.2021 ± 0.0194, p<0.001 , *d_z_* = 4.0133) (Fig. 5, right). These statistical differences between conditions were also true on an individual participant level for all participants. Success rates were also similar as in the out-of-scanner validation experiment, with an average torque response success rate of 95.10±5.71% , 50.29±29.76% , and 99.86±0.59% for Yield, Resist, and Slow conditions, respectively (Figs. 5 and 6).

#### 3.2.2 Brainstem activation

Brainstem-specific analysis revealed significant activity for Resist > 0, Resist > Yield, and Resist > Slow contrasts. Tables 2, 3, and 4 include the top three peaks of activation on the left and right sides of the brainstem, broken down by cluster and brainstem region. Notably, the contrasts Slow > 0, Yield > 0, and Yield > Slow did not result in any significant activation in the brainstem.

**Table 2.** Brainstem regions activation: Resist > 0.

Cluster	#Voxels	Region	Laterality	*p_FWE_*	x	y	z
Clust1	195	Midbrain	L	<0.0001	-4	-30	-12
			L	<0.0001	-8	-30	-8
			L	0.0002	-2	-30	-2
			R	0.0007	4	-30	-12
			R	0.0012	6	-28	-8
			R	0.0012	4	-28	-8
Clust2	133	Pons	L	0.0001	-6	-32	-46
			R	0.0034	2	-26	-44
			C	0.0043	0	-24	-46
			R	0.0246	4	-42	-38
			L	0.0266	-8	-28	-38
Clust1	185		L	<0.0001	-6	-30	-18
			R	0.0001	10	-28	-20
			L	0.0004	-4	-36	-24
			R	0.0005	4	-34	-18
			R	0.0010	12	-34	-22
			L	0.0040	-12	-34	-25
Clust2	259	Medulla	C	<0.0001	0	-38	-42
			L	<0.0001	-4	-40	-46
			L	<0.0001	-6	-36	-46
			R	<0.0001	6	-40	-50
			R	0.0001	8	-38	-46
			R	0.0002	4	-46	-62
			L	0.0008	-6	-38	-56

**Table 3.** Brainstem regions activation: Resist > Yield.

Cluster	#Voxels	Region	Laterality	*p_FWE_*	x	y	z
Clust1	95	Midbrain	L	0.0009	-8	-30	-4
			L	0.0010	-2	-30	-2
			R	0.0022	4	-30	-6
			L	0.0022	-6	-34	-4
			R	0.0038	8	-30	-2
Clust2	117	Pons	L	0.0005	-8	-42	-40
			L	0.0005	-8	-36	-44
			L	0.0008	-4	-40	-38
			R	0.0028	8	-40	-38
			R	0.0029	8	-34	-46
			R	0.0160	10	-36	-38
			C	0.0233	0	-28	-44
Clust1	174		L	0.0008	-10	-30	-20
			L	0.0010	-6	-36	-24
			L	0.0011	-4	-28	-20
			R	0.0030	12	-34	-22
			R	0.0048	10	-28	-20
			R	0.0048	4	-34	-26
Clust2	174	Medulla	C	<0.0001	0	-38	-42
			R	0.0003	2	-36	-46
			R	0.0004	8	-38	-46
			L	0.0004	-6	-40	-42
			R	0.0007	4	-44	-60
			C	0.0039	0	-42	-50
			L	0.0052	-4	-40	-56
			L	0.0054	-6	-44	-60

**Table 4.** Brainstem regions activation: Resist > Slow.

Cluster	#Voxels	Region	Laterality	*p_FWE_*	x	y	z
Clust1	89	Midbrain	L	0.0010	-4	-30	-12
			L	0.0028	-8	-30	-4
			L	0.0032	-2	-30	-2
			R	0.0057	2	-32	-4
			R	0.0086	8	-26	-16
			R	0.0087	6	-28	-14
	100	Pons	L	0.0006	-4	-32	-18
			L	0.0007	-10	-32	-20
			R	0.0008	2	-32	-18
			R	0.0025	10	-30	-18
			R	0.0067	8	-40	-22
			L	0.0173	-6	-36	-24
Clust2	50		C	0.0030	0	-34	-40
			L	0.0058	-8	-36	-42
			L	0.0112	-4	-42	-38
			R	0.0253	8	-40	-38
	29	Medulla	C	0.0025	0	-38	-42
Clust3	20		R	0.0270	8	-38	-48
			R	0.0270	8	-38	-46
			R	0.0321	6	-40	-52

**Fig. 7.**
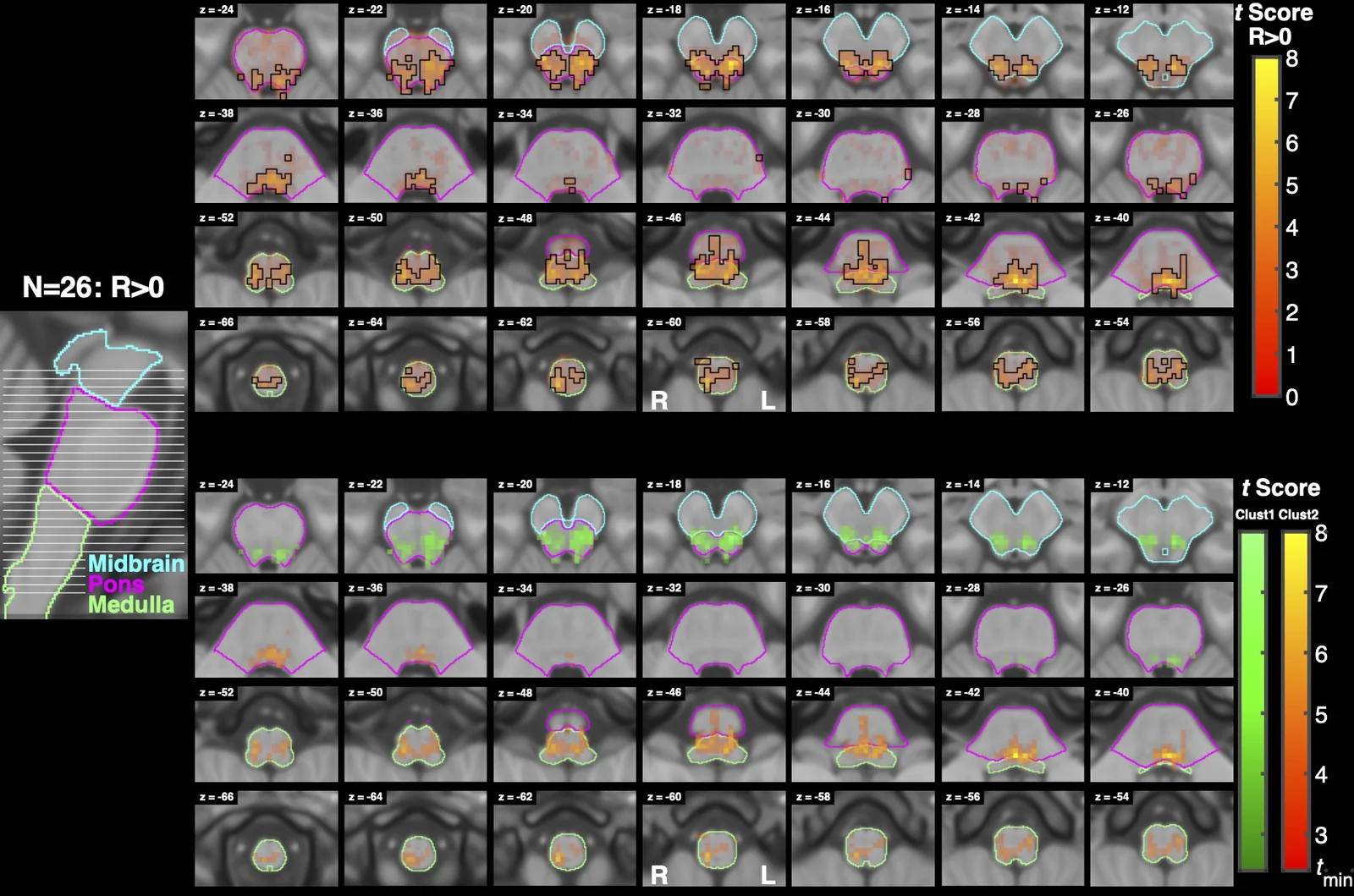
Activation associated with the Resist > 0 contrast. A sagittal slice on the left shows the location of each axial slice of the brainstem shown on the right with the highest slice on the top right and the lowest slice on the bottom left. Three sections of the brainstem are outlined with different colors to facilitate orientation. (Top) Functional maps for the contrast Resist > 0. Voxel color maps to *t* score (no lower limit), while opacity maps to TFCE significance, with significant voxels outlined in black. (Bottom) Cluster analysis of activation measured for the Resist > 0 contrast. Different clusters of significant activation are color coded, and voxel color is modulated by cluster and *t* score. For this analysis $t_{min}\;=2.4$ .

**Fig. 8.**
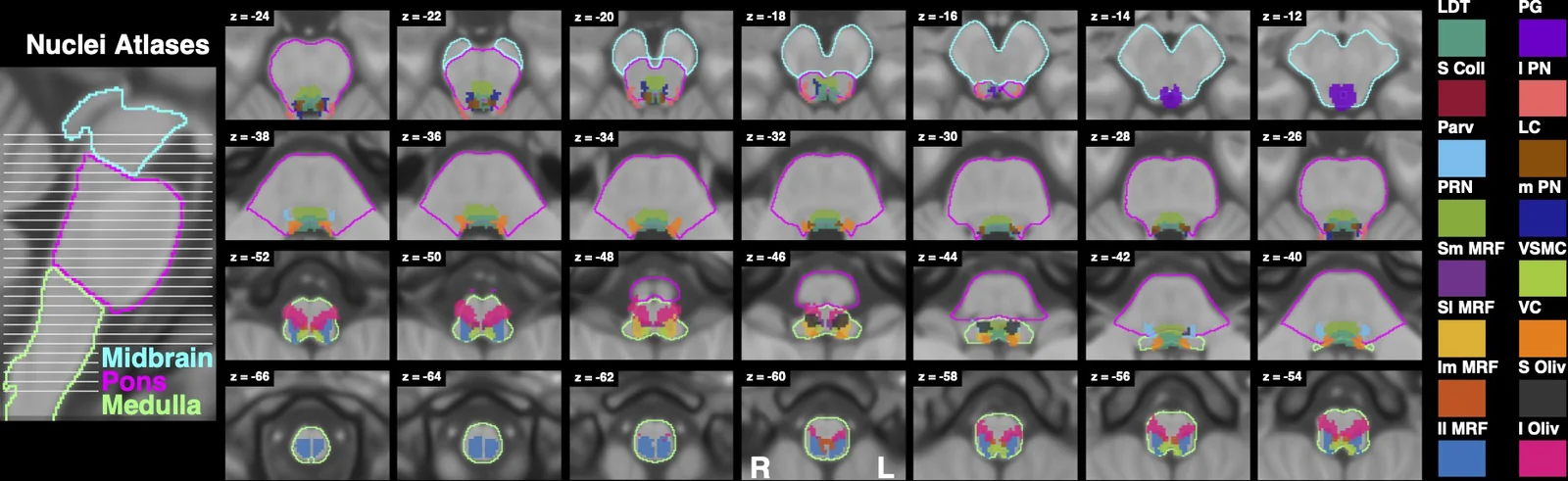
Brainstem slices with binarized atlases of referenced nuclei for comparison with functional activation figures. Abbreviations: laterodorsal tegmental nucleus (LDT), superior colliculus (S Coll), parvicellular reticular nucleus (Parv), pontine reticular nucleus (PRN), superior medial medullary reticular formation (Sm MRF), superior lateral medullary reticular formation (Sl MRF), inferior medial medullary reticular formation (Im MRF), inferior lateral medullary reticular formation (Il MRF), periaqueductal gray (PG), lateral parabrachial nucleus (l PN), locus coeruleus (LC), medial parabrachail nucleus (m PN), viscero-sensorimotor nuclei complex (VSMC), vestibular nuclei complex (VC), superior olivary complex (S Oliv), and inferior olivary nucleus (I Oliv). Conventions are the same as in Figure 7.

As shown in Figure 7 and Table 2, significant activity for Resist > 0 was widespread, with bilateral clusters spanning the midbrain, pons, and medulla. There were two clusters with more than five above-threshold voxels. The first cluster’s peaks spanned the midbrain and pons bilaterally, while the second cluster’s peaks spanned the pons and medulla bilaterally. The first cluster mostly overlapped bilaterally with the superior colliculi and periaqueductal gray and ipsilaterally with the pontine reticular nucleus. The second cluster mostly overlapped bilaterally with the lateral inferior medullary reticular formations, inferior olivary nuclei, and pontine reticular nuclei and contralaterally with the laterodorsal tegmental nucleus. A reference guide for nuclei locations is shown in Figure 8.

**Fig. 9.**
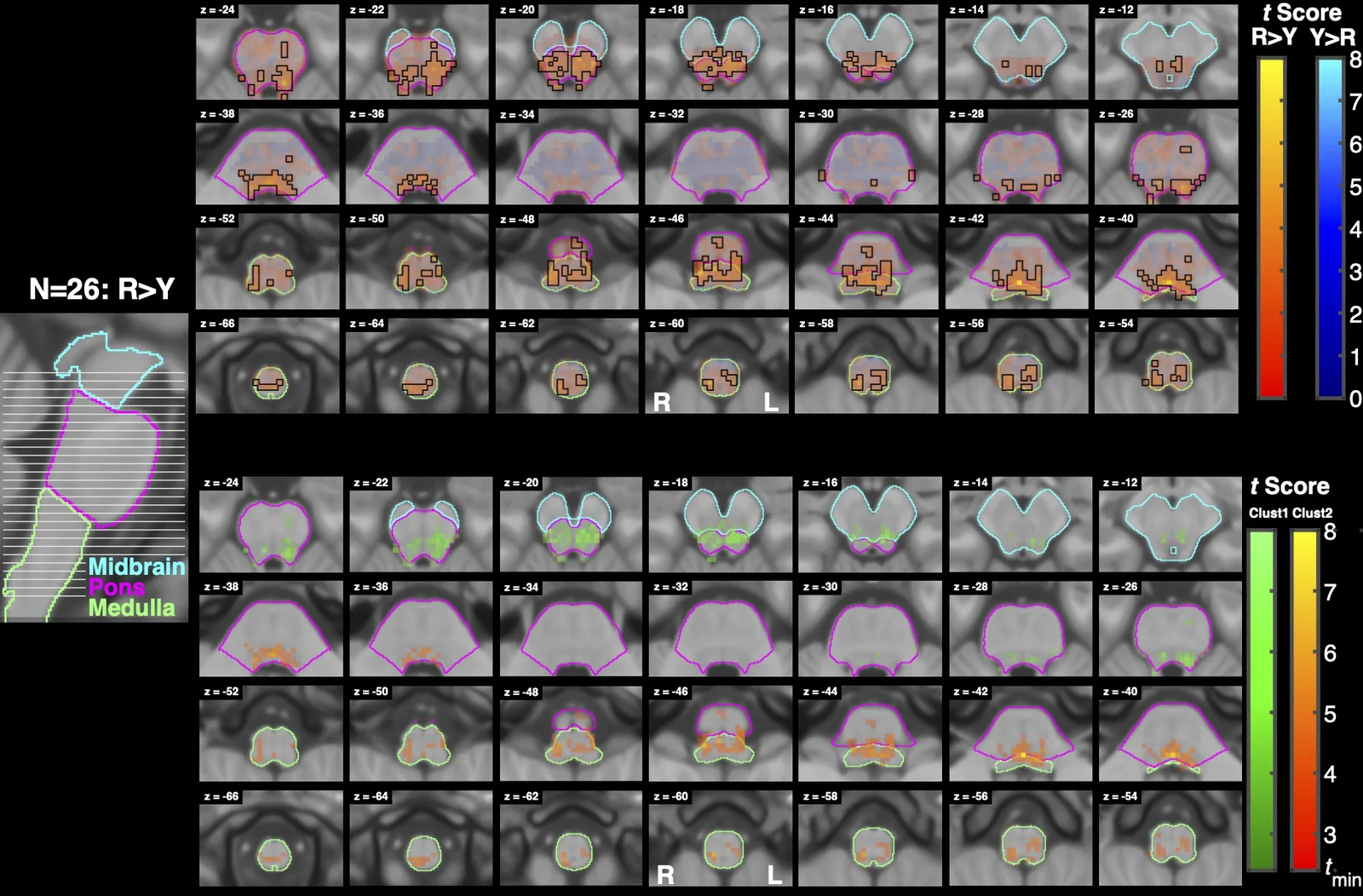
Activation associated with the Resist > Yield contrast. Conventions are the same as in Figure 7. (Top) Functional maps for the contrast Resist > Yield. (Bottom) Cluster analysis of activation measured for the contrast Resist > Yield. For this analysis $t_{min}\;=2.4$ .

**Fig. 10.**
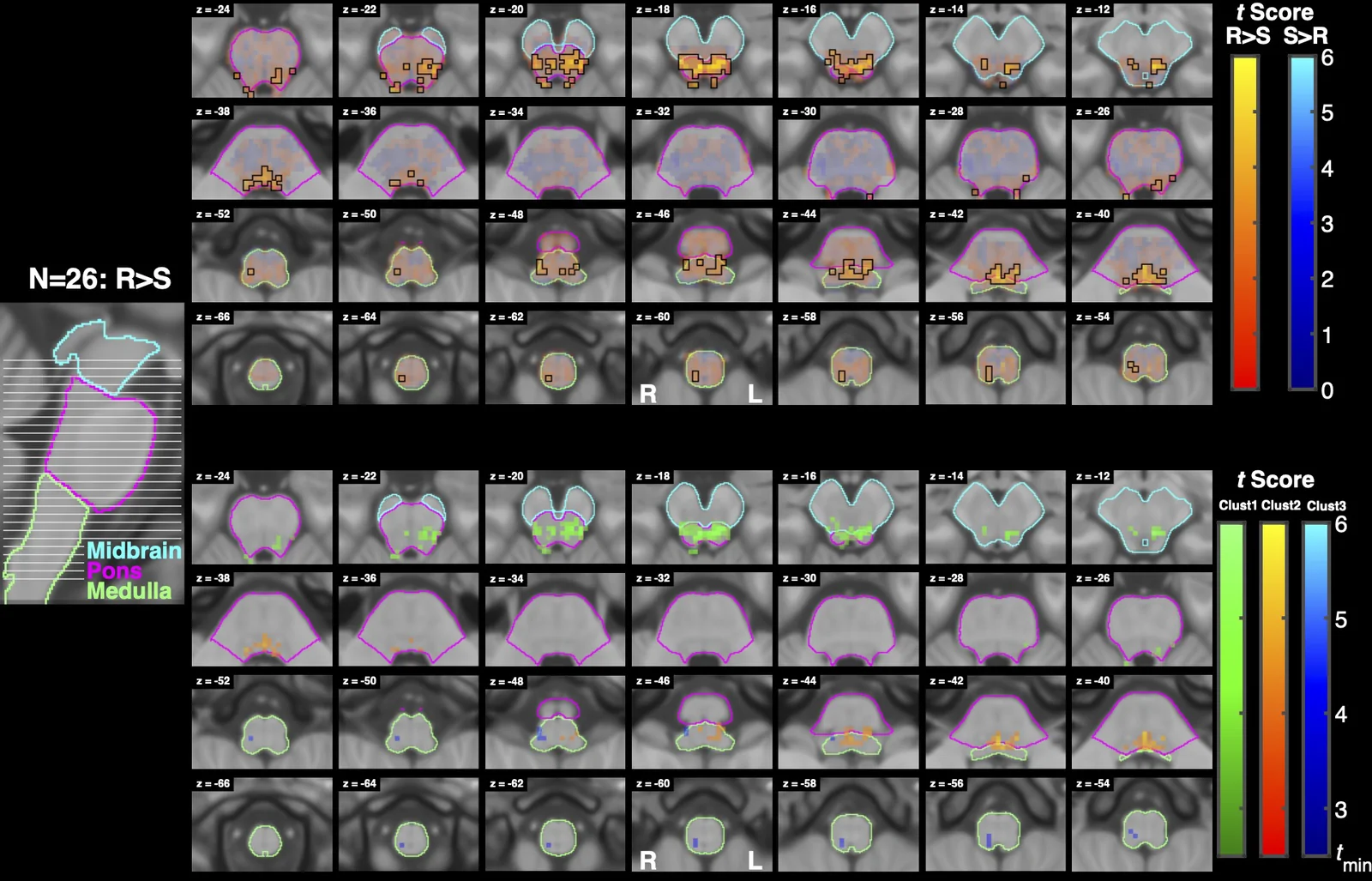
Activation associated with the Resist > Slow contrast. Conventions are the same as in Figure 7. (Top) Functional maps for the contrast Resist > Slow. (Bottom) Cluster analysis of activation measured for the contrast Resist > Slow. For this analysis $t_{min}\;=2.5$ .

Resist > Yield (Fig. 9) and Resist > Slow (Fig. 10) activity spanned similar locations as Resist > 0, and were also widespread, with clusters localizing bilaterally across the midbrain, pons, and medulla. The Resist > Yield contrast resulted in two clusters with more than five voxels. As with the Resist > 0 contrast, the first Resist > Yield cluster’s peaks spanned the midbrain and pons bilaterally, while the second cluster’s peaks spanned the pons and medulla bilaterally. The first cluster overlapped bilaterally with the pontine reticular nuclei, superior colliculi, and the periaqueductal gray. The second cluster mostly overlapped bilaterally with the lateral inferior medullary reticular formations and pontine reticular nuclei, and contralaterally with the inferior olivary nucleus and parvicellular reticular nucleus.

The Resist > Slow contrast resulted in three clusters with more than five voxels. The first cluster’s peaks spanned the midbrain and pons bilaterally, the second cluster’s peaks spanned the pons and medulla bilaterally, and the third cluster’s peaks spanned the ipsilateral medulla. The first cluster overlapped with the periaqueductal gray, bilateral superior colliculi, and ipsilateral pontine reticular nucleus. The second cluster mostly overlapped bilaterally with the pontine reticular nuclei, laterodorsal tegmental nuclei, and vetibular nuclei complexes, contralaterally with the parvicellular reticular nucleus, subcoeruleus, superior olivary complex, and medial and lateral superior medullary reticular formations. Finally, the third cluster mostly overlapped ipsilaterally with the superior and inferior lateral medullary reticular formations, superior and inferior olivary nuclei, viscero-sensory-motor nuclei complex, inferior medial medullary reticular formation, and parvicellular reticular nucleus.

**Fig. 11.**
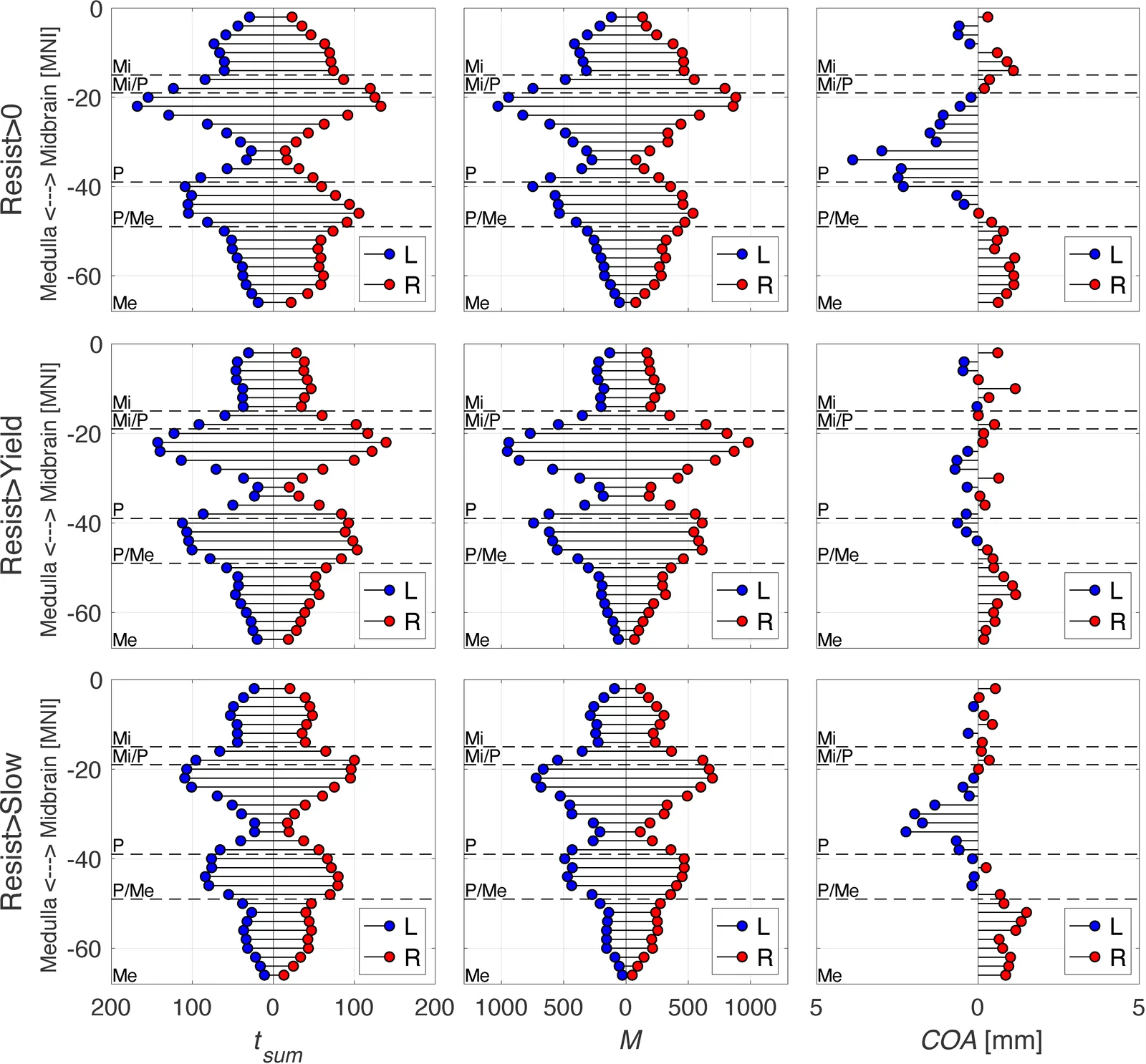
Laterality of positive activation in the brainstem. The three methods of quantifying laterality are shown with *t_sum_* on the left, *M* in the middle, and *COA* on the right with the contrasts Resist > 0 on the top, Resist > Yield in the middle, and Resist > Slow on the bottom. Outcome values are on each x axis and axial slice location (*z* slice in MNI coordinates) is on each y axis. Horizontal dashed lines separate different sections of the brainstem by medulla (Me), pons (P), midbrain (Mi), and their combinations. Blue dots indicate activity from the left (contralateral) hemisphere and red dots indicate activity from the right (ipsilateral) hemisphere. Right is ipsilateral and left is contralateral.

The results of our laterality analysis are summarized in Figure 11 for all contrasts of interest (R > 0, R > Y, R > S). All three outcomes of activation intensity (*t_sum_*), activation moment (*M*), and center of activation (COA) indicate a gradient of laterality of significant activation that changes from ipsilateral in medullar slices to contralateral in pontine slices, with no clear lateralization in the midbrain. Specifically, the center of activation is always lateralized ipsilaterally in medullar slices for all contrasts, while almost always lateralized contralaterally in pontine slices (always for R > 0 and R > S, in most slices for R > Y).

#### 3.2.3 Cortical activation

Whole-brain analysis revealed significant cortical activation for Slow > 0, Yield > 0, Resist > 0, Resist > Yield, and Resist > Slow contrasts.

Activation associated with the contrast Slow > 0 localized in the contralateral primary and secondary somatosensory cortices (S1, S2), premotor cortex (PM), and visual cortex (Brodmann Area 18). Additionally significant peaks localized in the ipsilateral visual cortex (Brodmann Area 17), inferior temporal gyrus, posterior supramarginal gyrus, and inferior parietal lobule. Finally, bilateral peaks localized in the primary motor cortices, inferior lateral occipital cortices, and superior parietal lobules.

Yield > 0 significant peaks were observed in the contralateral premotor cortex, primary motor cortex, primary and secondary somatosensory cortices, superior parietal lobule, visual cortex (Brodmann Area 18), insular cortex, and Broca’s Area 44, as well as the ipsilateral visual cortex (Brodmann Area 17) and anterior intraparietal sulcus hIP3.

**Fig. 12.**
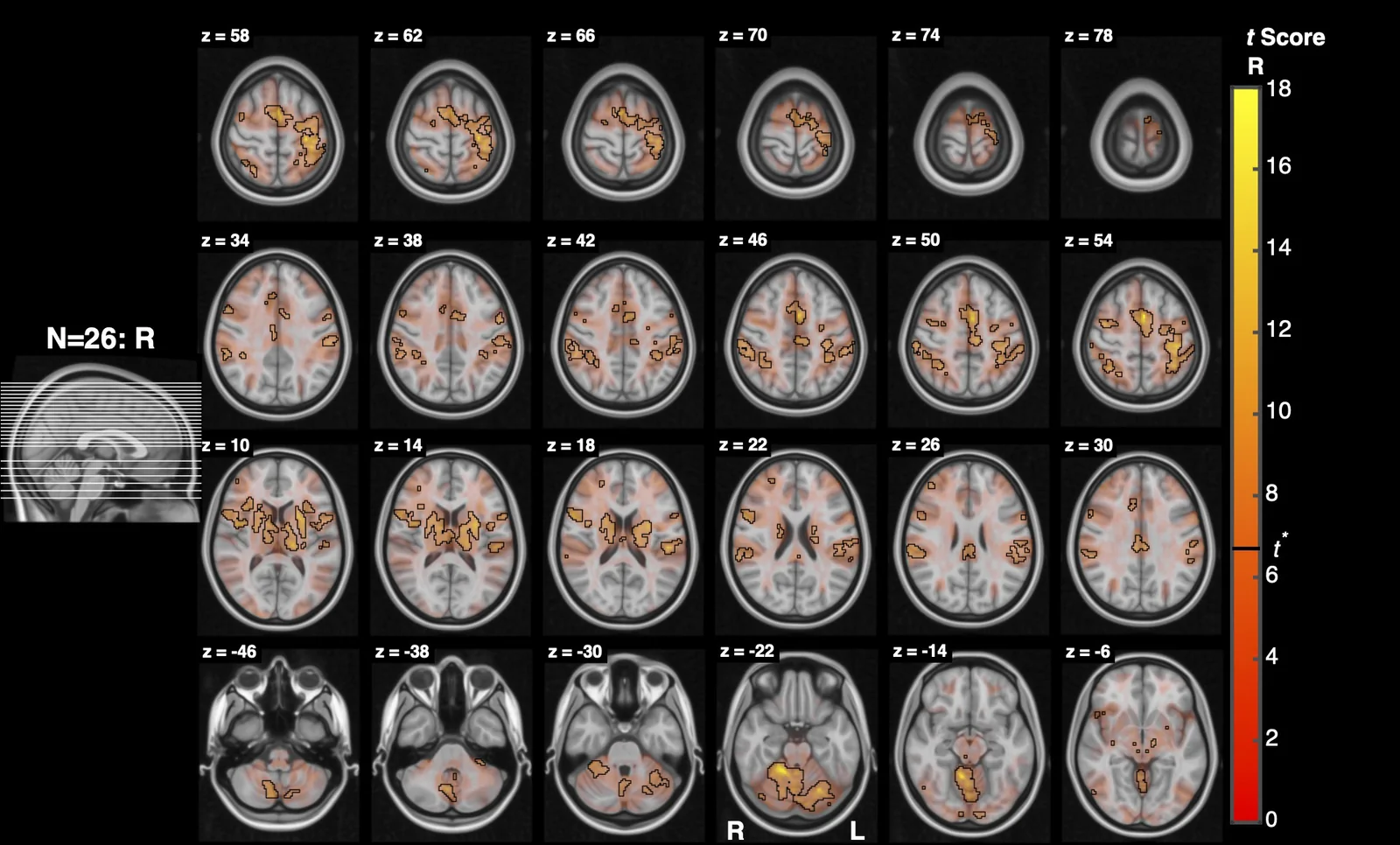
Cortical activation associated with the Resist > 0 contrast. A sagittal slice on the left shows the location of each axial slice of the brain shown on the right with the highest slice on the top right and the lowest slice on the bottom left. Functional activation is shown with lower t score voxels in red and higher t score voxels in yellow. Opacity is also modulated by t score and significant voxels are outlined in black.

Significant Resist > 0 peaks in the contralateral visual cortex (Brodmann Area 18), ipsilateral Broca’s Area 44, central opercular cortex, anterior intraparietal sulci HIP1 and hIP3, visual cortex V1, anterior supramarginal gyrus, parietal operculum cortex, superior parietal lobule 7A, frontal pole, paracingulate gyrus, and occipital pole, as well as bilateral peaks in the premotor cortices, visual cortex V5, and posterior cingulate gyri (Fig. 12).

**Fig. 13.**
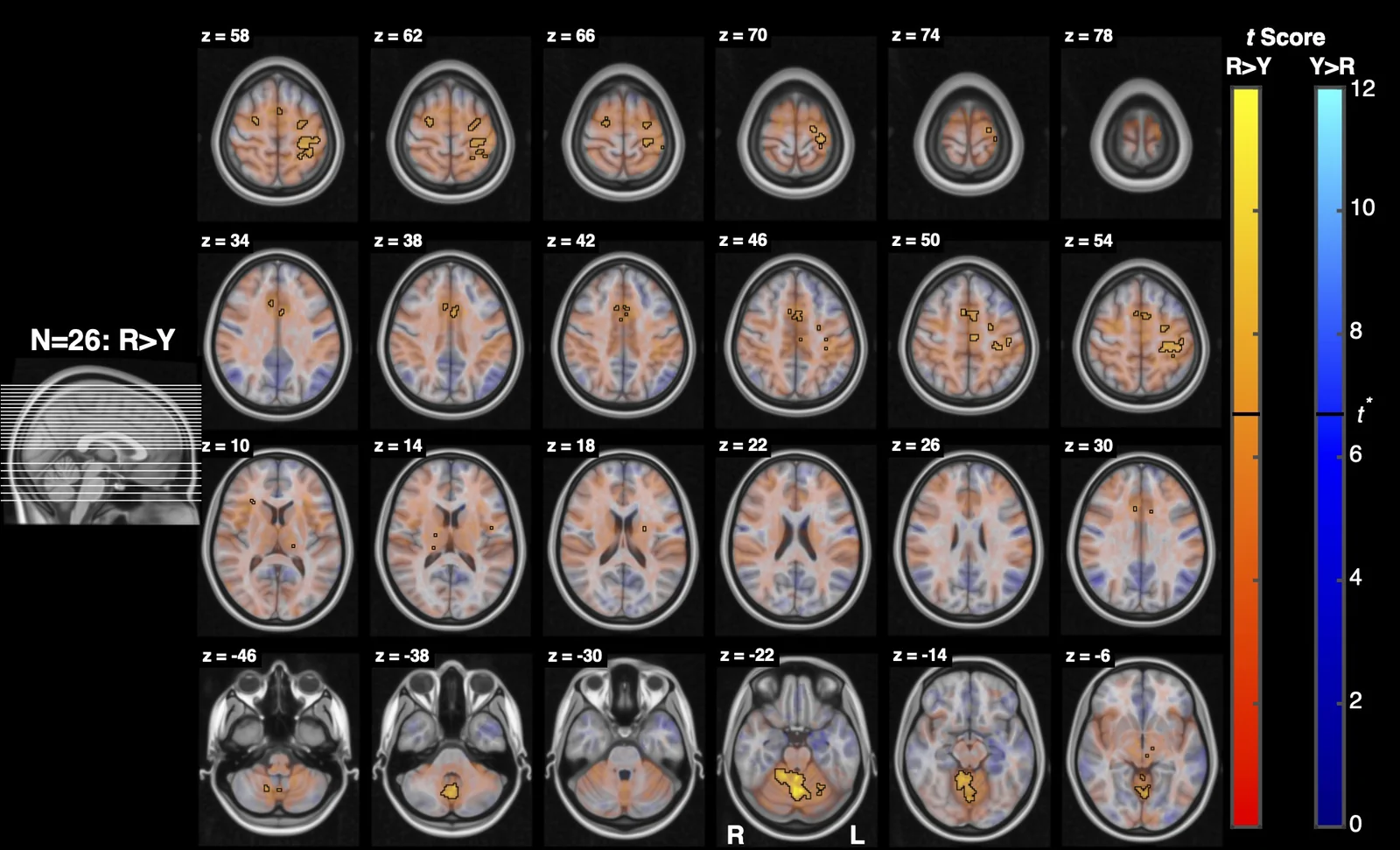
Cortical activation associated with the Resist > Yield contrast. Conventions are the same as in Figure 12.

Resist > Yield had significant peaks in the contralateral primary motor cortex, and primary somatosensory cortex, and bilateral anterior cingulate gyri, premotor cortices, and superior frontal gyri (Fig. 13).

**Fig. 14.**
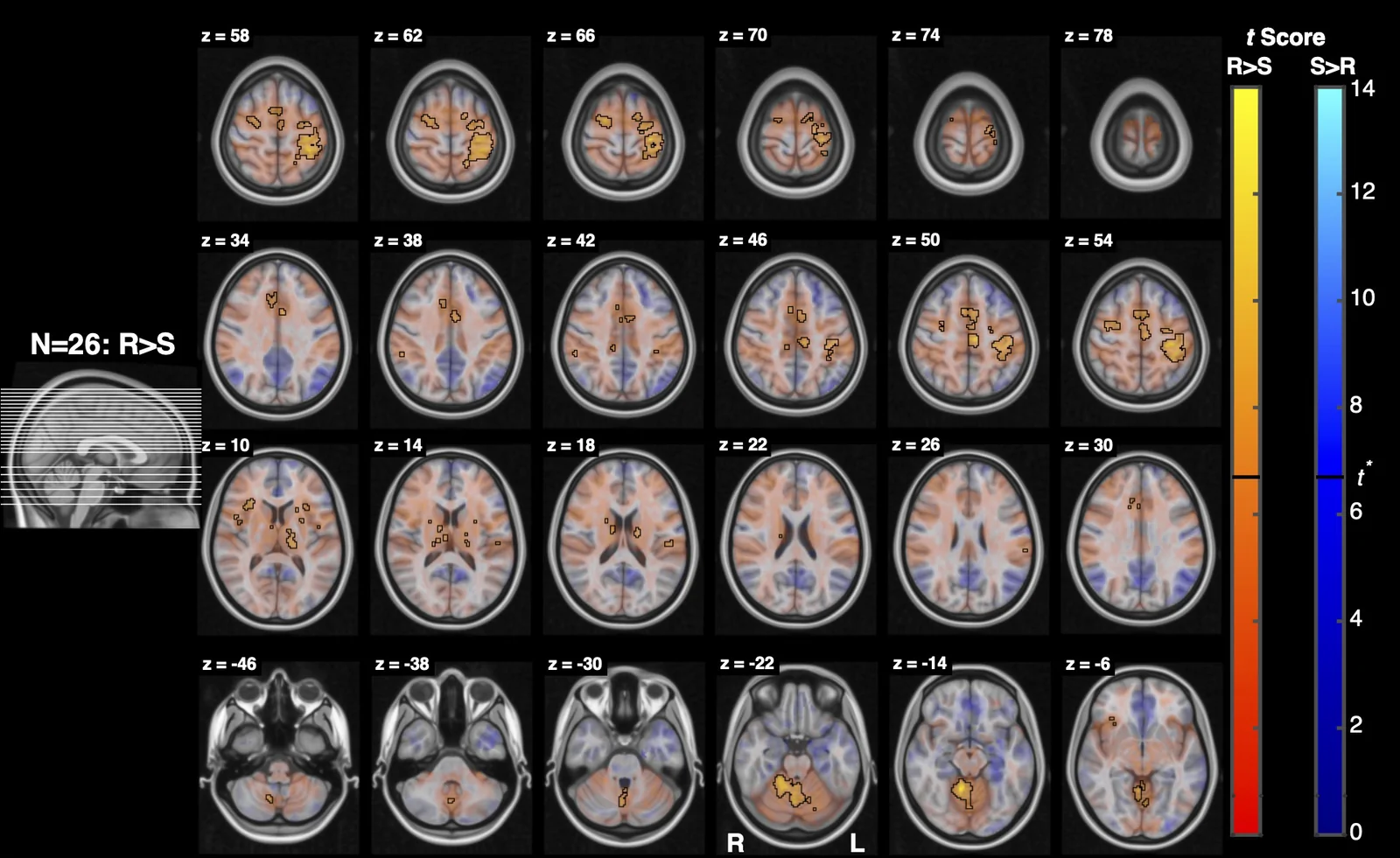
Cortical activation associated with the Resist > Slow contrast. Conventions are the same as in Figure 12.

Resist > Slow had significant contralateral peaks in the primary and secondary somatosensory cortices, anterior cingulate gyrus, and superior frontal gyrus. Ipsilateral peaks localized in the premotor cortex, frontal orbital cortex, frontal operculum cortex, central opercular cortex, posterior supramarginal gyrus, and posterior cingulate gyrus. Bilateral activity localized in the primary motor cortices, insular cortices, and Broca’s area 44 (Fig. 14).

#### 3.2.4 Subcortical and cerebellar activation

Whole-brain analysis revealed significant subcortical activation for Slow > 0, Yield > 0, Resist > 0, Resist > Yield, and Resist > Slow contrasts.

Significant peaks for Slow > 0 localized contralaterally in the thalamus and Crus I of the cerebellum, ipsilaterally in cerebellar lobes V, VIIb, and VIIIb, and bilaterally in the putamen and cerebellar lobe VI, as well as more centrally in lobe VI of the cerebellar vermis.

Yield > 0 had significant contralateral peaks in the thalamus and Crus I of the cerebellum and ipsilateral peaks in cerebellar lobes I-IV, VI, VIIb, VIIIa, and VIIIb.

Resist > 0 had significant contralateral peaks in the thalamus, putamen, and cerebellar lobes VI, VIIb, and X, ipsilateral peaks in cerebellar lobe V and Crus I, and bilateral peaks in cerebellar lobe VIIIa.

Resist > Yield had significant contralateral peaks in the thalamus and cerebellar lobes V and VI, ipsilateral peaks in cerebellar lobes I–IV and V, as well as more centrally located peaks in lobes VIIIa and IX of the cerebellar vermis.

Finally, Resist > Slow had significant contralateral peaks in the putamen and cerebellar lobe VI, ipsilateral peaks in cerebellar lobes V and VIIIb, and bilateral peaks in the thalamus.

## 4 Discussion

This study sought to determine whether human brainstem activity, particularly within brainstem regions consistent with the reticulospinal system, contributes to task-dependent modulation of stretch-evoked motor responses. Specifically, we tested whether instructing participants to resist a wrist perturbation would increase reticular formation activation relative to yielding, and whether the spatial organization of that activation would exhibit a rostrocaudal laterality gradient consistent with the double reciprocal model of reticulospinal control. Using high-resolution whole-brain fMRI optimized for the brainstem combined with a perturbation paradigm known to modulate long-latency responses, we quantified differences in BOLD activity under separate instructions and assessed laterality across brainstem slices.

### 4.1 H1: Task-dependent modulation of brainstem activity

Our first hypothesis predicts that stretch-evoked activity in the RF will be greater when participants are instructed to “resist” a perturbation than when instructed to “yield”. When resisting a perturbation, feedback gains are upregulated and prior work suggests that this increase cannot be fully explained by spinal or cortical circuits alone and instead, it reflects descending control from subcortical structures including the RST (Ravichandran et al., 2013; Shemmell et al., 2009). The current results support this hypothesis: the Resist > Yield contrast (Fig. 8) revealed significant bilateral activation throughout the pons and medulla, including regions consistent with the pontine and medullary reticular nuclei, inferior olivary nucleus, and parvicellular reticular nucleus. These areas overlap with atlas-defined regions containing nuclei that are considered primary motor origins of the RST, supporting the interpretation that task-dependent modulation of feedback responses involves reticulospinal-related recruitment (Kurtzer, 2014; Peterson, 1979; Pruszynski & Scott, 2012).

The observed activation in the Resist > Yield contrast overlaps regions containing nuclei with well-established roles in postural control, sensorimotor integration, and muscle tone regulation. The pontine reticular, gigantocellular, and parvicellular reticular nuclei have well-established origins of reticulospinal projections involved in regulating proximal limb muscle activity during reaching (Fisher et al., 2021; Riddle et al., 2009; Schepens & Drew, 2004). Additionally, although classically associated with cerebellar-driven motor learning, the inferior olivary nucleus also participates in timing and error-detection processes that influence response modulation. Prior work suggests inferior olivary nucleus involvement in adapting feedback responses (Llinás, 2009; Mundorf et al., 2025; Zeeuw et al., 1998). The periaqueductal gray, although not traditionally considered a group of motor nuclei, regulates arousal, multisensory integration, and orienting behaviors. Its activation here may reflect modulation of readiness or gain setting during the resist instruction rather than direct motor output (Koutsikou et al., 2015; Zhang et al., 2024).

Notably, the expected gradient of activation across Slow, Yield, and Resist conditions was less evident than anticipated. Both the Yield > 0 and Slow > 0 contrasts produced minimal brainstem activation, resulting in Resist > Yield and Resist > Slow contrasts that were similar in spatial extent and magnitude (Figs. 8 and 9). This observation suggests that task instructions may engage the brainstem more categorically than continuously. Rather than scaling linearly with effort or compliance, the transition to a “resist” task instruction may engage a relatively full reticulospinal command, with limited intermediate differentiation between Yield and Slow conditions. Under this interpretation, the Resist condition recruits a broader descending command to upregulate feedback gains, whereas Yield and Slow may rely more heavily on automatic sensorimotor feedback processes mediated cortically through long-latency reflex pathways, with comparatively limited additional brainstem involvement (Kurtzer, 2014; Perenboom et al., 2015).

Alternatively, the Yield condition may have relied more heavily on spinal feedback mechanisms with minimal supraspinal involvement leading to weaker brainstem differentiation between Slow and Yield conditions. Our EMG experiment resulted in LLR amplitudes that were not statistically different between Slow and Yield conditions, though there was a qualitatively notable change during the LLR window. Thus, it is possible that either our study was methodologically insensitive or our lack of a strong Yield > Slow contrast could reflect reduced RST contribution in those conditions.

The behavioral and EMG findings support this interpretation. Although torque production followed the expected ordering (Resist > Yield > Slow), the distinction between Yield and Slow was modest. In particular, LLRa was not significantly different between Yield and Slow, whereas Resist selectively enhanced LLRa relative to Yield. Moreover, SLRa showed clearer modulation between Yield and Slow, suggesting that spinal mechanisms may account for much of the differentiation between these two conditions. Together, these findings align with the imaging results, indicating that substantial reticulospinal engagement may be preferentially associated with the Resist instruction.

These findings differ in part with our previous StretchfMRI study (Zonnino et al., 2021), which reported significant activation in the medullary and pontine reticular formation during perturbations performed with a “yield” instruction. While the present study similarly detected stretch-related activity in the brainstem, we did not observe significant activation for the Yield condition alone (see Supplementary Fig. S16).

Methodological differences from our earlier StretchfMRI study may help explain the discrepancy. Critically, Zonnino et al. modeled brainstem activity as scaling with trial-by-trial EMG amplitude, identifying regions in which BOLD signal covaried with the magnitude of the evoked muscle response. In contrast, the present study examined condition-level main effects relative to baseline, without explicitly testing for parametric relationships with EMG. These analytic approaches probe related but distinct questions: one tests whether brainstem activity tracks the magnitude of motor output, whereas the other tests whether overall activation differs between task instructions. Additionally, the prior study employed an event-related design rather than a block design, which may have differentially influenced habituation or the separation of stretch-related and non-stretch-related activity. The present study also incorporated physiological noise regression and multi-echo denoising procedures, which may have reduced false positives in small subcortical regions and yielded a more conservative estimate of stretch-related activation.

### 4.2 H2: Brainstem activations show a rostrocaudal lateralization gradient that is consistent with the double reciprocal model of brainstem motor control

Our findings provide evidence that task instruction modulates activity in regions associated with the reticulospinal system, underscoring the brainstem’s role as a dynamic contributor to goal-dependent feedback control. The bilateral distribution of activation across the pons and medulla suggests that both medial and lateral RST pathways are involved, consistent with their known bilateral projections (Baker, 2011; Buford, 2009). Importantly, the spatial pattern of activation reveals a structured organization that aligns with aspects of the double reciprocal model of the RF while also highlighting complexities beyond a simple two-tract framework.

The double reciprocal model proposes complementary roles for the descending RST pathways: the medial RST provides excitatory drive to contralateral extensor muscles and inhibitory signal to contralateral flexor muscles, while the lateral RST provides excitatory drive to ipsilateral flexor muscles and inhibitory signal to ipsilateral extensor muscles (Davidson & Buford, 2006; Davidson et al., 2004, 2007; Peterson et al., 1978; Sakai et al., 2009). Recordings in non-human primates suggest general organizational tendencies along rostrocaudal and mediolateral axes, although these studies also emphasize the heterogeneity and bilateral branching of reticulospinal neurons. In this context, the model provides a useful framework for interpreting large-scale trends in activation patterns. Our results align with aspects of this organization but also highlight the predominantly diffuse and bilateral nature of brainstem activity during goal-dependent feedback control.

Across all three contrasts with significant brainstem activity, laterality analyses revealed a consistent ipsilateral-leaning pattern within the medulla, shifting toward a more bilateral or slightly contralateral-leaning pattern at the pontomedullary interface, and ultimately transitioning to contralateral-dominant activity in the pons, particularly in the Resist > 0 and Resist > Slow contrasts. This inferior–superior gradient mirrors the expected anatomical arrangement. The lateral RST, originating in the medulla, has stronger ipsilateral projections, while the medial RST, originating in the pons, has more bilateral/contralateral influence. The fact that the medulla consistently shows ipsilateral-biased activation while the pons shifts contralateral supports the interpretation that these activation patterns are consistent with the known anatomical organization of the reticulospinal system.

Importantly, the overall gradient is stable across methods for computing laterality. However, only the Resist > Slow contrast demonstrated strong unilateral dominance in the medulla. The gradient is thus more of a modest directional trend superimposed on a fundamentally bilateral system. The magnitudes of these laterality effects are modest. The *COA* in the medulla is consistently ipsilateral across contrasts, but the displacement is small, typically on the order of 1–2 mm. Similarly, laterality assessed using *t_sum_* reveals only moderate asymmetries. Even in the most lateralized contrast (Resist > Slow), the ipsilateral *t_sum_* in medullary slices is at most 150% of the contralateral value, indicating a bias rather than unilateral dominance. Pontine slices show the opposite directional trend, but again with relatively small spatial shifts and magnitude differences. Thus, although the direction of the gradient is reproducible across laterality metrics (*t_sum_*, *M*, and *COA*), the absolute degree of hemispheric separation remains limited.

Thus, rather than providing strong support for the double reciprocal model, our findings suggest (1) spatial differentiation along the pons/medulla boundary is present, mirroring the anatomical origins of the medial and lateral RSTs, (2) laterality changes gradually, not categorically, indicating that both pathways are likely co-activated during goal-dependent feedback responses, and (3) functional specialization may exist, but in humans it likely operates within an integrated and bilateral framework.

Functionally, resisting a perturbation requires both flexor responses to counteract the imposed stretch and extensor responses to stabilize the limb, which likely explains the simultaneous engagement of medullary and pontine nuclei. The bilateral nature of the responses is also expected. The RST is known to support whole-body postural adjustments and primate reticulospinal neurons frequently show bilateral or branching projections (Davidson & Buford, 2006).

### 4.3 Whole-brain imaging results

Whole-brain analyses revealed robust activation across a distributed sensorimotor network for all task conditions, including M1, S1, S2, PM, and superior parietal regions. These areas are known to support proprioceptive processing and corrective motor responses during limb perturbations (Omrani et al., 2016; Scott, 2004), confirming that the paradigm reliably engaged core sensorimotor circuits.

Yield > 0 and Resist > 0 showed broader recruitment of premotor and parietal regions than Slow > 0, consistent with the greater demands on sensorimotor integration when participants intentionally modulated their feedback responses (Pruszynski & Scott, 2012).

The contrasts Resist > Yield and Resist > Slow highlighted increased activation in PM, cingulate, and frontal opercular areas that are regions associated with effort, action monitoring, and rapid updating of motor commands (Rushworth et al., 2004). Stronger S1 and M1 responses in these contrasts align with the larger corrective torques and enhanced long-latency responses observed behaviorally and via EMG.

Subcortically, activation in cerebellar sensorimotor regions (lobules V, VI, VIII) increased significantly for all contrasts except Yield > Slow. These regions contribute to online error correction and the integration of proprioceptive information to update motor commands and shape long-latency feedback responses (Manto et al., 2012; Zonnino et al., 2021).

In addition to cerebellar activation, whole-brain analyses revealed putamen and thalamic involvement across task conditions. Putamen activation was observed bilaterally in the Slow > 0 contrast and contralaterally in both Resist > 0 and Resist > Slow contrasts, suggesting a contralateral-dominant but bilateral pattern consistent with its role in upper limb motor control. The putamen is a central component of cortico-basal ganglia circuits involved in the execution and parametric control of voluntary movement, including the regulation of movement scaling and vigor, and is known to integrate proprioceptive input to modulate ongoing motor output rather than initiate it (DeLong & Strick, 1974; Shirinbayan et al., 2019; Turner & Desmurget, 2010). Its engagement across conditions is, therefore, consistent with sustained voluntary contraction demands and the modulation of motor output during interaction with external loads rather than condition-specific differences between Yield and Resist.

The thalamus, which exhibited predominantly contralateral activation across all task conditions and bilateral activation in the Resist > Slow contrast, serves as a key relay between both cerebello-thalamo-cortical and basal ganglia-thalamo-cortical circuits. Our findings are consistent with those of Suminski et al. (2007), who also reported thalamic activation during robotic wrist perturbations and demonstrated that activity tracked moment-by-moment position errors rather than force production. Together, these findings support the interpretation that thalamic activation reflects integration of proprioceptive and cerebellar error-related information to support online motor corrections, while also relaying basal ganglia output during voluntary motor engagement (Bosch-Bouju et al., 2013; Butler et al., 1998).

Notably, Resist > Yield did not result in robust putamen or thalamic activation, suggesting that while these subcortical structures contribute to general motor execution and feedback integration across tasks, they are less directly involved in the task-dependent modulation of LLRs. This distinction supports the interpretation that such modulation may instead rely more heavily on brainstem pathways.

Finally, cortical maps for the Resist > Yield and Resist > Slow contrasts qualitatively, though not significantly, showed patterns consistent with reduced activation in regions typically associated with the default mode network, suggesting that the Resist condition shifts processing toward task-related demands and away from internally focused activation. This interpretation is consistent with our understanding that there is task-related suppression of default mode network activation during cognitively demanding tasks (Buckner et al., 2008).

### 4.4 Muscle activity and torque performance

Experiment 1 confirmed that participants produced distinct neuromuscular responses across the three tasks. As expected, the tasks produced clearly different levels of torque: Resist generated the largest amount of torque, Yield produced less, and the Slow condition elicited a minimal response. This same ordering appeared in Experiment 2. These parallel behavioral outcomes suggest that participants successfully understood and executed the task under both experimental environments.

The EMG results collected in Experiment 1 further validate that the perturbation paradigm elicits instruction-dependent modulation of LLRa, consistent with previous studies (Lewis et al., 2006; Pruszynski et al., 2008; Weiler et al., 2015). Importantly, Resist background activity was not larger than that of Yield or Slow, indicating that the greater torque produced during Resist relied more heavily on the fast feedback responses as opposed to elevated anticipatory muscle activation. The observed differences in SLRa and LLRa support this interpretation: both response epochs were facilitated in Resist compared with Slow, and LLRa was selectively enhanced in Resist compared with Yield.

Importantly, ECU EMG showed a modest but statistically significant effect of instruction on background activity. Participants exhibited elevated ECU activity during Resist compared with that during both Yield and Slow conditions, which may reflect increased co-contraction when preparing for a more challenging task. Greater antagonist activation could contribute to increased joint impedance via changes in muscle short-range stiffness, representing a potential confound when interpreting task-dependent differences in evoked responses. However, the magnitude of this background modulation was smaller than the observed modulation of LLRa, suggesting that changes in anticipatory activation along are unlikely to account for the enhanced LLRa during Resist.

### 4.5 Limitations and future directions

Interpreting activity in the brainstem warrants caution. Brainstem fMRI is constrained by the small size of brainstem nuclei, high vascular density, and sensitivity to motion and physiological noise, all of which reduce spatial specificity even under optimized acquisition pipelines. Many of the nuclei considered in this study have dimensions on the order of only a few millimeters in plane (Supplementary Table S5), comparable with the 1.731 × 1.731 × 4 mm^3^ spatial resolution of our functional images. Consequently, while our activation patterns overlap with atlas-defined reticular formation regions and other brainstem nuclei, the present data do not have definitive localization to individual nuclei. Our findings should, therefore, be interpreted as evidence of reticulospinal involvement rather than anatomically precise activation of specific nuclei.

Although our design successfully isolated task-dependent differences between our three conditions, an important limitation is the absence of a no-perturbation or slow-perturbation control matched to Resist. As a result, we cannot fully dissociate neural activity related to the intention to resist or increased motor readiness or arousal from activity driven by the stretch-evoked feedback response itself. The increased engagement required during Resist trials may recruit preparatory and arousal-related networks in addition to pathways involved in processing the stretch response. Therefore, the observed brainstem activation should not be interpreted as exclusively reflecting reflex modulation. Future experiments incorporating a Resist Slow condition will be necessary to better separate the contributions of stretch-evoked responses from task-related motor preparation.

The present block design was selected to maximize statistical power, as block designs provide increased sensitivity for detecting low-amplitude brainstem signals, which are inherently challenging to measure with fMRI. This increased sensitivity was prioritized to establish the feasibility of detecting task-dependent modulation of brainstem activation during perturbations. However, block designs provide less flexibility for independently modeling closely related cognitive and motor processes, such as anticipatory motor preparation and stretch-evoked responses. Incorporating a Resist Slow condition would require an event-related design in which perturbation types are interleaved, allowing these processes to be more directly examined while minimizing differences in task expectation between conditions. Future studies building upon this approach may leverage an event-related design to further characterize the relative contributions of motor preparation, arousal, and stretch-evoked responses to the observed response. Furthermore, because fMRI measures hemodynamic responses integrated over several seconds rather than millisecond-scale neural events, observed activation cannot be attributed exclusively to the timing of the reflex response itself. Rather, these signals likely reflect the combined neural processes engaged during preparation, perturbation processing, and the resulting motor response.

Finally, we did not record forces from the non-perturbed (left) wrist. Without bilateral measurements, we cannot determine whether participants generated coordinated or symmetric torque responses, which could explain the bilateral functional imaging results. Future studies incorporating bilateral force sensors and a Resist condition with a slow perturbation would help clarify the specific contributions to intention, feedback, and bilateral coordination to task-dependent modulation.

## 5 Conclusion

This study demonstrates that goal-dependent modulation of rapid feedback responses is associated with measurable changes in human brainstem activity. Using an MRI-compatible robotic wrist perturbation paradigm combined with a brainstem-optimized multi-echo whole-brain fMRI protocol, we identified bilateral activation within pontine and medullary regions consistent with major reticulospinal nuclei during a Resist instruction relative to Yield. Although activation patterns were largely diffuse and bilateral, laterality analyses revealed a modest rostrocaudal gradient consistent with known anatomical trends in reticulospinal organization. Importantly, behavioral and EMG results confirmed instruction-dependent modulation of long-latency responses, supporting the interpretation that descending pathways contribute to the observed imaging differences.

The absence of a clear activation gradient across Slow, Yield, and Resist conditions suggests that reticulospinal engagement may operate in a relatively categorical manner once a resistive motor set is adopted, rather than scaling linearly with task demands. At the same time, the diffuse and bilateral nature of the activation patterns underscores the distributed and multisynaptic organization of the human brainstem motor system.

More broadly, these findings establish the feasibility of integrating MRI-compatible robotics, physiological monitoring, and advanced denoising strategies to probe brainstem motor circuits noninvasively. This approach provides a foundation for future studies investigating descending control in health and disease, including stroke, spinal cord injury, and motor rehabilitation, where reticulospinal pathways are thought to play a critical compensatory role.

## Supplementary Material

Supplementary Material

## Data Availability

Data and code are publicly available on OpenNeuro at doi:10.18112/openneuro.ds008159.v1.0.1 and GitHub at https://github.com/rnikon/process_fMRI_yieldresist.git.

## References

[IMAG.a.1372-b1] Amunts, K., Mohlberg, H., Bludau, S., & Zilles, K. (2020). Julich-Brain: A 3D probabilistic atlas of the human brain’s cytoarchitecture. Science, 369(6506), 988–992. 10.1126/science.abb4588 32732281

[IMAG.a.1372-b2] Baker, S. N. (2011). The primate reticulospinal tract, hand function and functional recovery. Journal of Physiology, 589(23), 5603–5612. 10.1113/jphysiol.2011.215160 PMC3249036 21878519

[IMAG.a.1372-b3] Bianciardi, M., Strong, C., Toschi, N., Edlow, B. L., Fischl, B., Brown, E. N., Rosen, B. R., & Wald, L. L. (2018). A probabilistic template of human mesopontine tegmental nuclei from in vivo 7 T MRI. NeuroImage, 170(April 2017), 222–230. 10.1016/j.neuroimage.2017.04.070 PMC5670016 28476663

[IMAG.a.1372-b4] Bianciardi, M., Toschi, N., Edlow, B. L., Eichner, C., Setsompop, K., Polimeni, J. R., Brown, E. N., Kinney, H. C., Rosen, B. R., & Wald, L. L. (2015). Toward an in vivo neuroimaging template of human brainstem nuclei of the ascending arousal, autonomic, and motor systems. Brain Connectivity, 5(10), 597–607. 10.1089/brain.2015.0347 PMC4684653 26066023

[IMAG.a.1372-b5] Bosch-Bouju, C., Hyland, B. I., & Parr-Brownlie, L. C. (2013). Motor thalamus integration of cortical, cerebellar and basal ganglia information: Implications for normal and parkinsonian conditions. Frontiers in Computational Neuroscience, 7(November), 163. 10.3389/fncom.2013.00163 PMC3822295 24273509

[IMAG.a.1372-b6] Buckner, R. L., Andrews-Hanna, J. R., & Schacter, D. L. (2008). The brain’s default network: Anatomy, function, and relevance to disease. Annals of the New York Academy of Sciences, 1124, 1–38. 10.1196/annals.1440.011 18400922

[IMAG.a.1372-b7] Buford, J. A. (2009). Reticulospinal system. In L. R. Squire (Ed.), Encyclopedia of neuroscience (Vol. 8, pp. 151–158). Academic Press. 10.1016/B978-008045046-9.01337-1

[IMAG.a.1372-b8] Butler, E. G., Harvey, M. C., Finkelstein, D. I., & Horne, M. K. (1998). Neuronal activity in the monkey ventrolateral thalamus following perturbations of voluntary wrist movements. Experimental Brain Research, 118, 393–407. 10.1007/s002210050293 9497146

[IMAG.a.1372-b9] Capaday, C., Forget, R., Fraser, R., & Lamarre, Y. (1991). Evidence for a contribution of the motor cortex to the long-latency stretch reflex of the human thumb. The Journal of Physiology, 440, 243–255. 10.1113/jphysiol.1991.sp018706 PMC1180150 1804962

[IMAG.a.1372-b10] Carlsen, A. N., Maslovat, D., Lam, M. Y., Chua, R., & Franks, I. M. (2011). Considerations for the use of a startling acoustic stimulus in studies of motor preparation in humans. Neuroscience and Biobehavioral Reviews, 35(3), 366–376. 10.1016/j.neubiorev.2010.04.009 20466020

[IMAG.a.1372-b11] Cox, R. W. (1996). AFNI: Software for analysis and visualization of functional magnetic resonance neuroimages. Computers and Biomedical Research, 29, 162–173. 10.1006/cbmr.1996.0014 8812068

[IMAG.a.1372-b12] Davidson, A. G., & Buford, J. A. (2006). Bilateral actions of the reticulospinal tract on arm and shoulder muscles in the monkey: Stimulus triggered averaging. Experimental Brain Research, 173, 25–39. 10.1007/s00221-006-0374-1 16506008

[IMAG.a.1372-b13] Davidson, A. G., Buford, J. A., & Motor, J. A. B. (2004). Motor outputs from the primate reticular formation to shoulder muscles as revealed by stimulus-triggered averaging. Journal of Neurophysiology, 92, 83–95. 10.1152/jn.00083.2003 PMC2740726 15014106

[IMAG.a.1372-b14] Davidson, A. G., Schieber, M. H., & Buford, J. A. (2007). Bilateral spike-triggered average effects in arm and shoulder muscles from the monkey pontomedullary reticular formation. Journal of Neuroscience, 27(30), 8053–8058. 10.1523/JNEUROSCI.0040-07.2007 PMC6672715 17652596

[IMAG.a.1372-b15] DeLong, M. R., & Strick, P. L. (1974). Relation of basal ganglia, cerebellum, and motor cortex units to ramp and ballistic limb movements. Brain Research, 71, 327–335. 10.1016/0006-8993(74)90975-5 4219745

[IMAG.a.1372-b16] Desikan, R. S., Ségonne, F., Fischl, B., Quinn, B. T., Dickerson, B. C., Blacker, D., Buckner, R. L., Dale, A. M., Maguire, R. P., Hyman, B. T., Albert, M. S., & Killiany, R. J. (2006). An automated labeling system for subdividing the human cerebral cortex on MRI scans into gyral based regions of interest. NeuroImage, 31(3), 968–980. 10.1016/j.neuroimage.2006.01.021 16530430

[IMAG.a.1372-b17] Diedrichsen, J., Balsters, J. H., Cussans, E., & Ramnani, N. (2009). A probabilistic MR atlas of the human cerebellum. NeuroImage, 46(1), 39–46. 10.1016/j.neuroimage.2009.01.045 19457380

[IMAG.a.1372-b18] Fisher, K. M., Zaaimi, B., Edgley, S. A., & Baker, S. N. (2021). Extensive cortical convergence to primate reticulospinal pathways. Journal of Neuroscience, 41(5), 1005–1018. 10.1523/JNEUROSCI.1379-20.2020 PMC7880280 33268548

[IMAG.a.1372-b19] Forgaard, C. J., Franks, I. M., Bennett, K., Maslovat, D., & Chua, R. (2018). Mechanical perturbations can elicit triggered reactions in the absence of a startle response. Experimental Brain Research, 236(2), 365–379. 10.1007/s00221-017-5134-x 29151141

[IMAG.a.1372-b20] Forgaard, C. J., Franks, I. M., Maslovat, D., Gowan, N. J., Kim, J. C., & Chua, R. (2016). An examination of the startle response during upper limb stretch perturbations. Neuroscience, 337, 163–176. 10.1016/j.neuroscience.2016.09.010 27664458

[IMAG.a.1372-b21] Frazier, J. A., Chiu, S., Breeze, J. L., Makris, N., Lange, N., Kennedy, D. N., Herbert, M. R., Bent, E. K., Koneru, V. K., Dieterich, M. E., Hodge, S. M., Rauch, S. L., Grant, P. E., Cohen, B. M., Seidman, L. J., Caviness, V. S., & Biederman, J. (2005). Structural brain magnetic resonance imaging of limbic and thalamic volumes in pediatric bipolar disorder. American Journal of Psychiatry, 162(7), 1256–1265. 10.1176/appi.ajp.162.7.1256 15994707

[IMAG.a.1372-b22] García-Gomar, M. G., Strong, C., Toschi, N., Singh, K., Rosen, B. R., Wald, L. L., & Bianciardi, M. (2019). In vivo probabilistic structural atlas of the inferior and superior colliculi, medial and lateral geniculate nuclei and superior olivary complex in humans based on 7 Tesla MRI. Frontiers in Genetics, 10(July), 1–15. 10.3389/fnins.2019.00764 PMC6694208 31440122

[IMAG.a.1372-b23] García-Gomar, M. G., Videnovic, A., Singh, K., Stauder, M., Lewis, L. D., Wald, L. L., Rosen, B. R., & Bianciardi, M. (2022). Disruption of brainstem structural connectivity in REM sleep behavior disorder using 7 Tesla magnetic resonance imaging. Movement Disorders, 37(4), 847–853. 10.1002/mds.28895 PMC9018552 34964520

[IMAG.a.1372-b24] Goldstein, J. M., Seidman, L. J., Makris, N., Ahern, T., O’Brien, L. M., Caviness, V. S. J., Kennedy, D. N., Faraone, S. V., & Tsuang, M. T. (2007). Hypothalamic abnormalities in schizophrenia: Sex effects and genetic vulnerability. Biological Psychiatry, 61(8), 935–945. 10.1016/j.biopsych.2006.08.027 17046727

[IMAG.a.1372-b25] Honeycutt, C. F., & Perreault, E. J. (2012). Planning of ballistic movement following stroke: Insights from the startle reflex (B. Draganski, Ed.). PLoS One, 7(8), e43097. 10.1371/journal.pone.0043097 PMC3431358 22952634

[IMAG.a.1372-b26] Iglesias, J. E., Van Leemput, K., Bhatt, P., Casillas, C., Dutt, S., Schuff, N., Truran-Sacrey, D., Boxer, A., & Fischl, B. (2015). Bayesian segmentation of brainstem structures in MRI. NeuroImage, 113, 184–195. 10.1016/j.neuroimage.2015.02.065 PMC4434226 25776214

[IMAG.a.1372-b27] Jenkinson, M., Beckmann, C. F., Behrens, T. E. J., Woolrich, M. W., & Smith, S. M. (2012). FSL. NeuroImage, 62, 782–790. 10.1016/j.neuroimage.2011.09.015 21979382

[IMAG.a.1372-b28] Kasper, L., Bollmann, S., Diaconescu, A. O., Hutton, C., Heinzle, J., Iglesias, S., Hauser, T. U., Sebold, M., Manjaly, Z. M., Pruessmann, K. P., & Stephan, K. E. (2017). The PhysIO toolbox for modeling physiological noise in fMRI data. Journal of Neuroscience Methods, 276, 56–72. 10.1016/j.jneumeth.2016.10.019 27832957

[IMAG.a.1372-b29] Kim, J. H., Taylor, A. J., Himmelbach, M., Hagberg, G. E., Scheffler, K., & Ress, D. (2022). Characterization of the blood oxygen level dependent hemodynamic response function in human subcortical regions with high spatiotemporal resolution. Frontiers in Neuroscience, 16, 1009295. 10.3389/fnins.2022.1009295 PMC9592726 36303946

[IMAG.a.1372-b30] Kourtis, D., Kwok, H. F., Roach, N., Wing, A. M., & Praamstra, P. (2008). Maintaining grip: Anticipatory and reactive EEG responses to load perturbations. Journal of Neurophysiology, 99, 545–553. 10.1152/jn.01112.2006 18032560

[IMAG.a.1372-b31] Koutsikou, S., Watson, T. C., Crook, J. J., Leith, J. L., Lawrenson, C. L., Apps, R., & Lumb, X. B. M. (2015). The periaqueductal gray orchestrates sensory and motor circuits at multiple levels of the neuraxis. Journal of Neuroscience, 35(42), 14132–14147. 10.1523/JNEUROSCI.0261-15.2015 PMC4683682 26490855

[IMAG.a.1372-b32] Kurtzer, I. L. (2014). Long-latency reflexes account for limb biomechanics through several supraspinal pathways. Frontiers in Integrative Neuroscience, 8, 99. 10.3389/fnint.2014.00099 PMC4310276 25688187

[IMAG.a.1372-b33] Lewis, G. N., Mackinnon, C. D., & Perreault, E. J. (2006). The effect of task instruction on the excitability of spinal and supraspinal reflex pathways projecting to the biceps muscle. Experimental Brain Research, 174, 413–425. 10.1007/s00221-006-0475-x PMC2756617 16676166

[IMAG.a.1372-b34] Llinás, R. R. (2009). Inferior olive oscillation as the temporal basis for motricity and oscillatory reset as the basis for motor error correction. Neuroscience, 162, 797–804. 10.1016/j.neuroscience.2009.04.045 PMC2861300 19393291

[IMAG.a.1372-b35] Makris, N., Goldstein, J. M., Kennedy, D., Hodge, S. M., Caviness, V. S., Faraone, S. V., Tsuang, M. T., & Seidman, L. J. (2006). Decreased volume of left and total anterior insular lobule in schizophrenia. Schizophrenia Research, 83(2–3), 155–171. 10.1016/j.schres.2005.10.016 16448806

[IMAG.a.1372-b36] Manto, M., Bower, J. M., Conforto, A. B., Delgado-García, J. M., da Guarda, S. N. F., Gerwig, M., Habas, C., Hagura, N., Ivry, R. B., Mariën, P., Molinari, M., Naito, E., Nowak, D. A., Taib, N. O. B., Pelisson, D., Tesche, C. D., Tilikete, C., & Timmann, D. (2012). Consensus paper: Roles of the cerebellum in motor control—The diversity of ideas on cerebellar involvement in movement. Cerebellum, 11, 457–487. 10.1007/s12311-011-0331-9 PMC4347949 22161499

[IMAG.a.1372-b37] Marsden, C. D., Merton, P. A., Morton, H. B., Hallett, M., Adam, J., & Rushton, D. N. (1977). Disorders of movement in cerebellar disease in man. In F. Rose (Ed.), The physiological aspect of clinical neurology (pp. 179–199). Blackwell. https://lccn.loc.gov/78316445

[IMAG.a.1372-b38] McPherson, J. G., Chen, A., Ellis, M. D., Yao, J., Heckman, C. J., & Dewald, J. P. (2018). Progressive recruitment of contralesional cortico-reticulospinal pathways drives motor impairment post stroke. Journal of Physiology, 596(7), 1211–1225. 10.1113/JP274968 PMC5878212 29457651

[IMAG.a.1372-b39] Medina, M. C., Reddy, N. A., Sitek, K. R., & Bright, M. G. (2025). Mapping whole-brain auditory activation with 3T multi-echo fMRI at the group and individual-subject level. bioRxiv. 10.64898/2025.12.03.692160 PMC13042257 41895044

[IMAG.a.1372-b40] Mundorf, A., Rice, L. C., Peterburs, J., & Desmond, J. E. (2025). Dynamic inferior olive activation in a cognitive task: An fMRI study. Brain Structure and Function, 230(5), 1–10. 10.1007/s00429-025-02933-5 PMC12095361 40397071

[IMAG.a.1372-b41] Nikonowicz, R. C., & Sergi, F. (2024). A novel experimental protocol for studying the task-dependent contribution of the brainstem to long-latency responses via MRI-compatible robotics. In Proceedings of the 10th IEEE RAS and EMBS International Conference on Biomedical Robotics and Biomechatronics (BioRob), September (pp. 1283–1288). IEEE. 10.1109/BioRob60516.2024.10719728

[IMAG.a.1372-b42] Omrani, M., Murnaghan, C. D., Pruszynski, J. A., & Scott, S. H. (2016). Distributed task-specific processing of somatosensory feedback for voluntary motor control. eLife, 5, e13141. 10.7554/eLife.13141 PMC4876645 27077949

[IMAG.a.1372-b43] Palmer, E., & Ashby, P. (1992). Evidence that a long latency stretch reflex in humans is transcortical. The Journal of Physiology, 449, 429–440. 10.1113/jphysiol.1992.sp019094 PMC1176087 1522516

[IMAG.a.1372-b44] Perenboom, M. J. L., Ruit, M. V. D., Groot, J. H. D., Schouten, A. C., & Meskers, C. G. M. (2015). Evidence for sustained cortical involvement in peripheral stretch reflex during the full long latency reflex period. Neuroscience Letters, 584, 214–218. 10.1016/j.neulet.2014.10.034 25449867

[IMAG.a.1372-b45] Peterson, B. W. (1979). Reticulospinal projections to spinal motor nuclei. Annual Review of Physiology, 41, 127–140. 10.1146/annurev.ph.41.030179.001015 373586

[IMAG.a.1372-b46] Peterson, B. W., Pitts, N. G., Fukushima, K., Mackel, R., Avenue, Y., & York, N. (1978). Brain reticulospinal excitation and inhibition of neck motoneurons. Experimental Brain Research, 32, 471–489. 10.1007/BF00239548 689126

[IMAG.a.1372-b47] Poldrack, R. A., Fletcher, P. C., Henson, R. N., Worsley, K. J., Brett, M., & Nichols, T. E. (2008). Guidelines for reporting an fMRI study. NeuroImage, 40, 409–414. 10.1016/j.neuroimage.2007.11.048 PMC2287206 18191585

[IMAG.a.1372-b48] Pruszynski, J. A., Kurtzer, I., Nashed, J. Y., Omrani, M., Brouwer, B., & Scott, S. H. (2011). Primary motor cortex underlies multi-joint integration for fast feedback control. Nature, 478(7369), 387–390. 10.1038/nature10436 PMC4974074 21964335

[IMAG.a.1372-b49] Pruszynski, J. A., Kurtzer, I., & Scott, S. H. (2008). Rapid motor responses are appropriately tuned to the metrics of a visuospatial task. Journal of Neurophysiology, 100(1), 224–238. 10.1152/jn.90262.2008 18463184

[IMAG.a.1372-b50] Pruszynski, J. A., & Scott, S. H. (2012). Optimal feedback control and the long-latency stretch response. Experimental Brain Research, 218(3), 341–359. 10.1007/S00221-012-3041-8 22370742

[IMAG.a.1372-b51] Ravichandran, V. J., Honeycutt, C. F., Shemmell, J., & Perreault, E. J. (2013). Instruction-dependent modulation of the long-latency stretch reflex is associated with indicators of startle. Experimental Brain Research, 230, 59–69. 10.1007/s00221-013-3630-1 PMC3759548 23811739

[IMAG.a.1372-b52] Reddy, N. A., Clements, R. G., Brooks, J. C., & Bright, M. G. (2024). Simultaneous cortical, subcortical, and brainstem mapping of sensory activation. Cerebral Cortex, 34(6), bhae273. 10.1093/cercor/bhae273 PMC11212354 38940832

[IMAG.a.1372-b53] Reddy, N. A., Zvolanek, K. M., Moia, S., Caballero-Gaudes, C., & Bright, M. G. (2024). Denoising task-correlated head motion from motor-task fMRI data with multi-echo ICA. Imaging Neuroscience, 2, imag_2_00057. 10.1162/imag_a_00057 PMC11426116 39328846

[IMAG.a.1372-b54] Riddle, C. N., Edgley, S. A., & Baker, S. N. (2009). Direct and indirect connections with upper limb motoneurons from the primate reticulospinal tract. Journal of Neuroscience, 29(15), 4993–4999. 10.1523/JNEUROSCI.3720-08.2009 PMC2690979 19369568

[IMAG.a.1372-b55] Rushworth, M. F. S., Walton, M. E., Kennerley, S. W., & Bannerman, D. M. (2004). Action sets and decisions in the medial frontal cortex. Trends in Cognitive Sciences, 8(9), 410–417. 10.1016/j.tics.2004.07.009 15350242

[IMAG.a.1372-b56] Sakai, S. T., Davidson, A. G., & Buford, J. A. (2009). Reticulospinal neurons in the pontomedullary reticular formation of the monkey (*Macaca fascicularis*). Neuroscience, 163(4), 1158–1170. 10.1016/j.neuroscience.2009.07.036 PMC2760615 19631726

[IMAG.a.1372-b57] Schepens, B., & Drew, T. (2004). Independent and convergent signals from the pontomedullary reticular formation contribute to the control of posture and movement during reaching in the cat. Journal of Neurophysiology, 92, 2217–2238. 10.1152/jn.01189.2003 15175364

[IMAG.a.1372-b58] Scott, S. H. (2004). Optimal feedback control and the neural basis of volitional motor control. Nature Reviews Neuroscience, 5(July), 534–546. 10.1038/nrn1427 15208695

[IMAG.a.1372-b59] Seghier, M. L. (2008). Laterality index in functional MRI: Methodological issues. Magnetic Resonance Imaging, 26(5), 594–601. 10.1016/j.mri.2007.10.010 PMC2726301 18158224

[IMAG.a.1372-b60] Shemmell, J., Je, H. A., & Perreault, E. J. (2009). The differential role of motor cortex in stretch reflex modulation induced by changes in environmental mechanics and verbal instruction. Journal of Neuroscience, 29(42), 13255–13263. 10.1523/JNEUROSCI.0892-09.2009 PMC2791059 19846713

[IMAG.a.1372-b61] Shirinbayan, S. I., Dreyer, A. M., & Rieger, J. W. (2019). Cortical and subcortical areas involved in the regulation of reach movement speed in the human brain: An fMRI study. Human Brain Mapping, 40, 151–162. 10.1002/hbm.24361 PMC6865438 30251771

[IMAG.a.1372-b62] Singh, K., García-Gomar, M. G., & Bianciardi, M. (2021). Probabilistic atlas of the mesencephalic reticular formation, isthmic reticular formation, microcellular tegmental nucleus, ventral tegmental area nucleus complex, and caudal-rostral linear raphe nucleus complex in living humans from 7 Tesla Magnetic Reso. Brain Connectivity, 11(8), 613–623. 10.1089/brain.2020.0975 PMC8817713 33926237

[IMAG.a.1372-b63] Singh, K., Indovina, I., Augustinack, J. C., Nestor, K., García-Gomar, M. G., Staab, J. P., & Bianciardi, M. (2020). Probabilistic template of the lateral parabrachial nucleus, medial parabrachial nucleus, vestibular nuclei complex, and medullary viscero-sensory-motor nuclei complex in living humans from 7 Tesla MRI. Frontiers in Neuroscience, 13(January), 1–11. 10.3389/fnins.2019.01425 PMC6989551 32038134

[IMAG.a.1372-b64] Smith, S. M., & Nichols, T. E. (2009). Threshold-free cluster enhancement: Addressing problems of smoothing, threshold dependence and localisation in cluster inference. NeuroImage, 44, 83–98. 10.1016/j.neuroimage.2008.03.061 18501637

[IMAG.a.1372-b65] Soteropoulos, D. S., & Baker, S. N. (2020). Long-latency responses to a mechanical perturbation of the index finger have a spinal component. The Journal of Neuroscience, 40(20), 3933–3948. 10.1523/JNEUROSCI.1901-19.2020 PMC7219296 32245828

[IMAG.a.1372-b66] Soteropoulos, D. S., Williams, E. R., & Baker, S. N. (2012). Cells in the monkey ponto-medullary reticular formation modulate their activity with slow finger movements. Journal of Physiology, 590(16), 4011–4027. 10.1113/jphysiol.2011.225169 PMC3476645 22641776

[IMAG.a.1372-b67] Spieser, L., Aubert, S., & Bonnard, M. (2013). Involvement of SMAp in the intention-related long latency stretch reflex modulation: A TMS study. Neuroscience, 246, 329–341. 10.1016/j.neuroscience.2013.05.005 23673280

[IMAG.a.1372-b68] Suminski, A. J., Rao, S. M., Mosier, K. M., & Scheidt, R. A. (2007). Neural and electromyographic correlates of wrist posture control. Journal of Neurophysiology, 97, 1527–1545. 10.1152/jn.01160.2006. 17135464

[IMAG.a.1372-b69] Turner, R. S., & Desmurget, M. (2010). Basal ganglia contributions to motor control: A vigorous tutor. Current Opinion in Neurobiology, 20, 704–716. 10.1016/j.conb.2010.08.022 PMC3025075 20850966

[IMAG.a.1372-b70] Valls-Solé, J., Rothwell, J. C., Goulart, F., Cossu, G., & Muñoz, E. (1999). Patterned ballistic movements triggered by a startle in healthy humans. Journal of Physiology, 516(3), 931–938. 10.1111/j.1469-7793.1999.0931u.x PMC2269293 10200438

[IMAG.a.1372-b71] Weiler, J., Gribble, P. L., & Pruszynski, J. A. (2015). Goal-dependent modulation of the long-latency stretch response at the shoulder, elbow, and wrist. Journal of Neurophysiology, 114(6), 3242–3254. 10.1152/JN.00702.2015 PMC4686281 26445871

[IMAG.a.1372-b72] Weinman, J., Arfa-Fatollahkhani, P., Zonnino, A., Nikonowicz, R. C., & Sergi, F. (2021). Effects of perturbation velocity, direction, background muscle activation, and task instruction on long-latency responses measured from forearm muscles. Frontiers in Human Neuroscience, 15, 639773. 10.3389/fnhum.2021.639773 PMC8085277 33935670

[IMAG.a.1372-b73] Zeeuw, C. I. D., Simpson, J. I., Hoogenraad, C. C., Galjart, N., Koekkoek, S. K. E., & Ruigrok, T. J. H. (1998). Microcircuitry and function of the inferior olive. Trends in Neurosciences, 21, 391–400. 10.1016/s0166-2236(98)01310-1 9735947

[IMAG.a.1372-b74] Zhang, H., Zhu, Z., Ma, W.-X., Kong, L.-X., Yuan, P.-C., Bu, L.-F., Han, J., Huang, Z.-L., & Wang, Y.-Q. (2024). The contribution of periaqueductal gray in the regulation of physiological and pathological behaviors. Frontiers in Neuroscience, 18(April), 1380171. 10.3389/fnins.2024.1380171 PMC11034386 38650618

[IMAG.a.1372-b75] Zonnino, A., Farrens, A. J., Ress, D., & Sergi, F. (2019). StretchfMRI: A novel technique to quantify the contribution of the reticular formation to long-latency responses via fMRI. In Proceedings of the IEEE 16th International Conference on Rehabilitation Robotics (ICORR), 2019–June (pp. 1247–1253). IEEE. 10.1109/ICORR.2019.8779451 PMC7874541 31374800

[IMAG.a.1372-b76] Zonnino, A., Farrens, A. J., Ress, D., & Sergi, F. (2021). Measurement of stretch-evoked brainstem function using fMRI. Scientific Reports, 11(1), 1–21. 10.1038/s41598-021-91605-5 PMC8206209 34131162

