# Supplementary material for: fMRI reveals brainstem and cortical networks contribute to the instruction-dependent modulation of stretch-evoked motor responses
Source: Imaging Neurosci (Camb). 2026 Sep 18;4:IMAG.a.1372. doi: 10.1162/IMAG.a.1372 (PMC13591507; doi:10.1162/IMAG.a.1372)
Supplement: Supplementary file 1 [file IMAG.a.1372_supp.pdf]

Supplementary Material

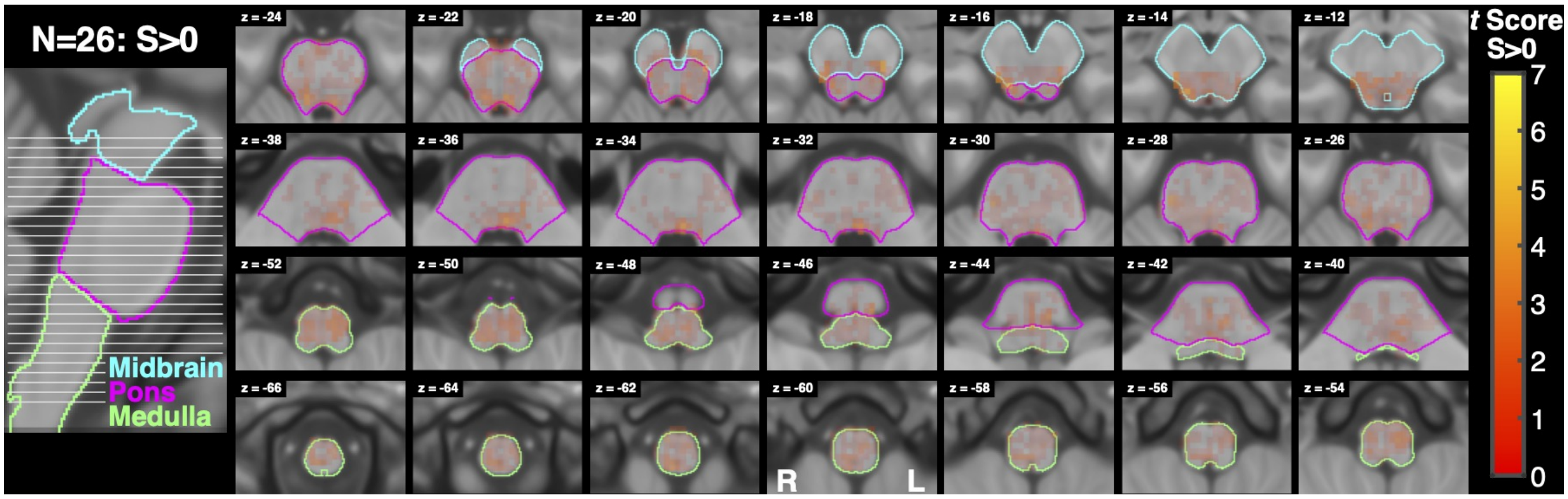

Figure 15: Activation associated with the Slow contrast. A sagittal slice on the left shows the location of each axial slice of the brainstem shown on the right with the highest slice on the top right and the lowest slice on the bottom left. Three sections of the brainstem are outlined with different colors to facilitate orientation. Voxel color maps to t score (no lower limit), while opacity maps to TFCE significance.

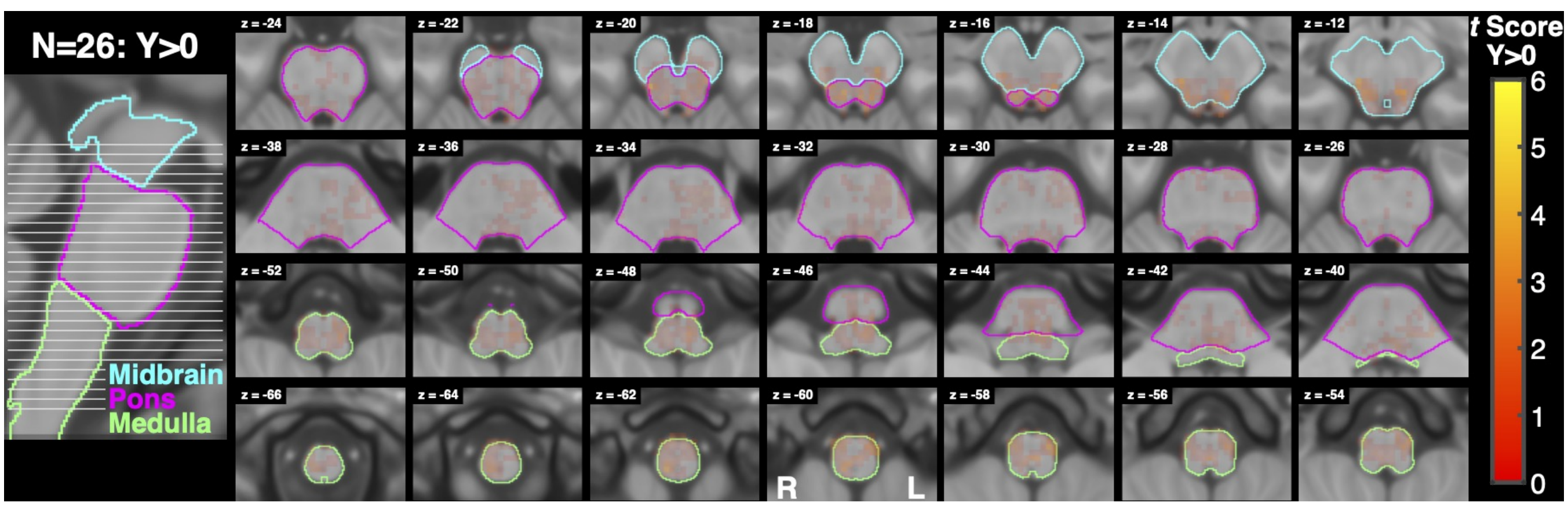

Figure 16: Activation associated with the Yield contrast. A sagittal slice on the left shows the location of each axial slice of the brainstem shown on the right with the highest slice on the top right and the lowest slice on the bottom left. Three sections of the brainstem are outlined with different colors to facilitate orientation. Voxel color maps to t score (no lower limit), while opacity maps to TFCE significance.

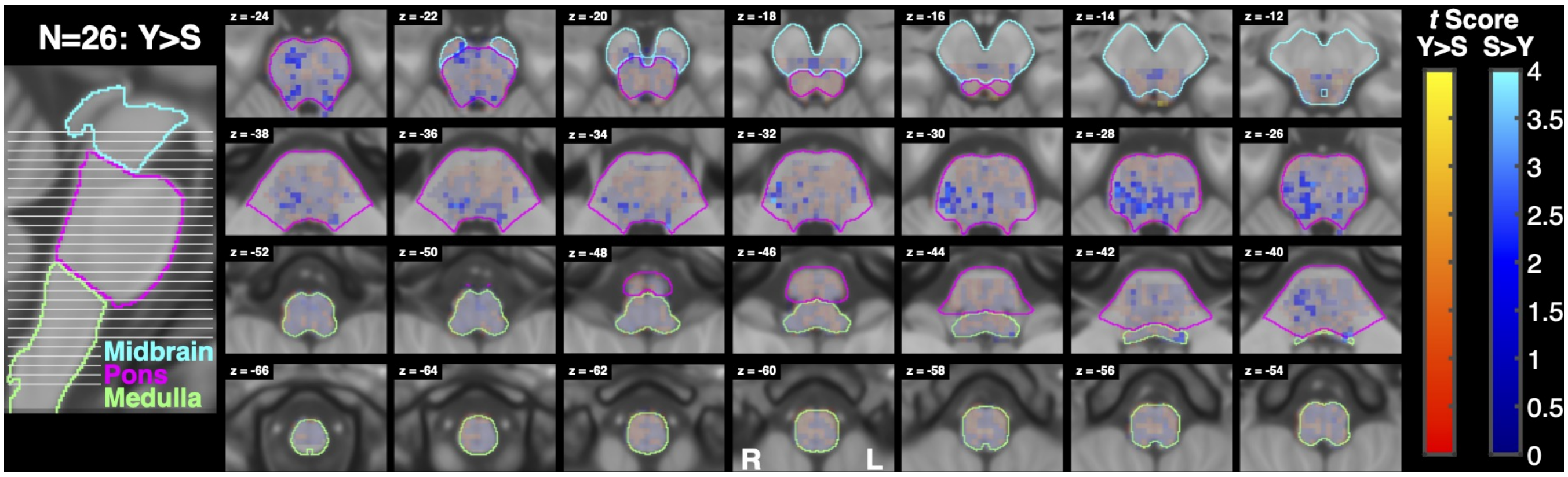

Figure 17: Activation associated with the Yield>Slow contrast. A sagittal slice on the left shows the location of each axial slice of the brainstem shown on the right with the highest slice on the top right and the lowest slice on the bottom left. Three sections of the brainstem are outlined with different colors to facilitate orientation. Voxel color maps to t score (no lower limit), while opacity maps to TFCE significance.

Table 5: Approximate dimensions of discussed brainstem nuclei. Dimensions were obtained from the Brainstem Navigator Atlas.

Brainstem Nucleus or Nuclei Cluster	Approximate Dimensions [mm]
Laterodorsal Tegmental Nucleus	6 × 6 × 28
Superior Colliculus	8 × 10 × 8
Parvicellular Reticular Nucleus	6 × 6 × 8
Pontine Reticular Nucleus	8 × 13 × 28
Superior Medial Medullary Reticular Formation	5 × 4 × 8
Superior Lateral Medullary Reticular Formation	8 × 9 × 7
Inferior Medial Medullary Reticular Formation	4 × 7 × 12
Inferior Lateral Medullary Reticular Formation	7 × 12 × 21
Periaqueductal Gray	10 × 15 × 16
Lateral Parabrachial Nucleus	4 × 12 × 13
Locus Coeruleus	6 × 6 × 14
Medial Parabrachial Nucleus	5 × 11 × 13
Viscero-Sensorimotor Nuclei Complex	5 × 9 × 16
Vestibular Nuclei Complex	7 × 9 × 18
Superior Olivary Complex	6 × 6 × 6
Inferior Olivary Nucleus	11 × 11 × 19

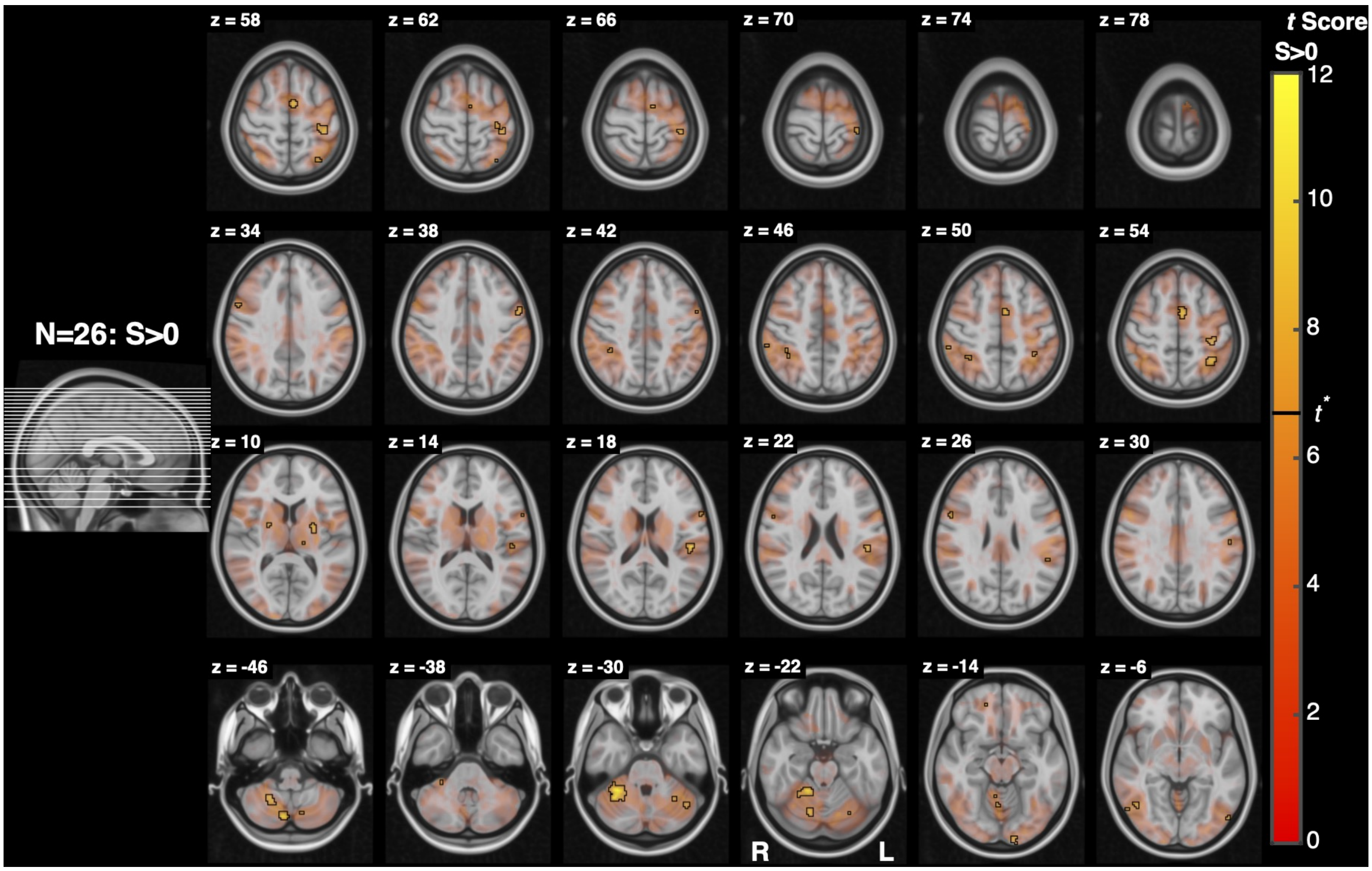

Figure 18: Cortical activation associated with the Slow contrast. A sagittal slice on the left shows the location of each axial slice of the brain shown on the right with the highest slice on the top right and the lowest slice on the bottom left. Functional activation is shown with lower t score voxels in red and higher t score voxels in yellow. Opacity is also modulated by t score and significant voxels are outlined in black.

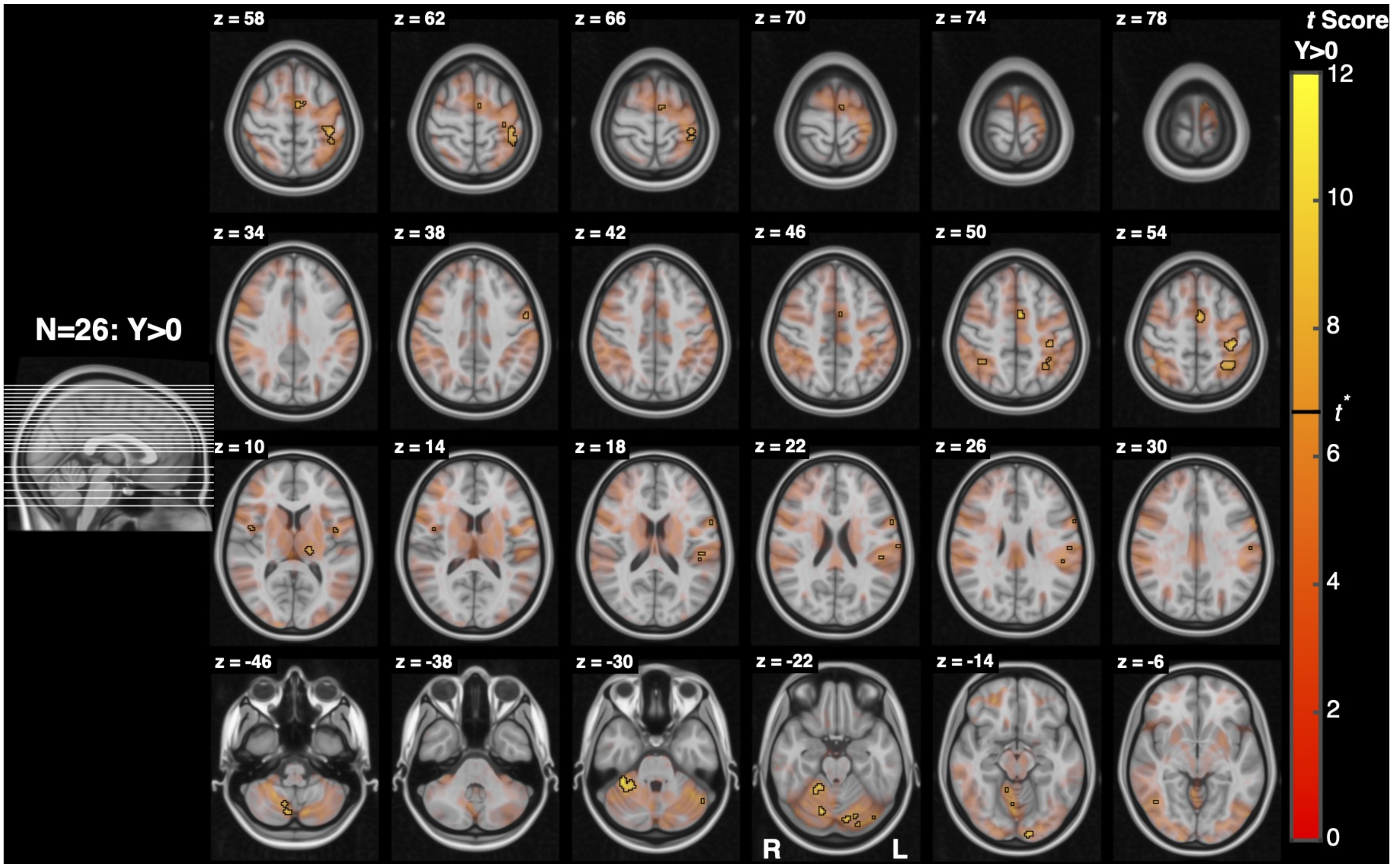

Figure 19: Cortical activation associated with the Yield contrast. A sagittal slice on the left shows the location of each axial slice of the brain shown on the right with the highest slice on the top right and the lowest slice on the bottom left. Functional activation is shown with lower t score voxels in red and higher t score voxels in yellow. Opacity is also modulated by t score and significant voxels are outlined in black.

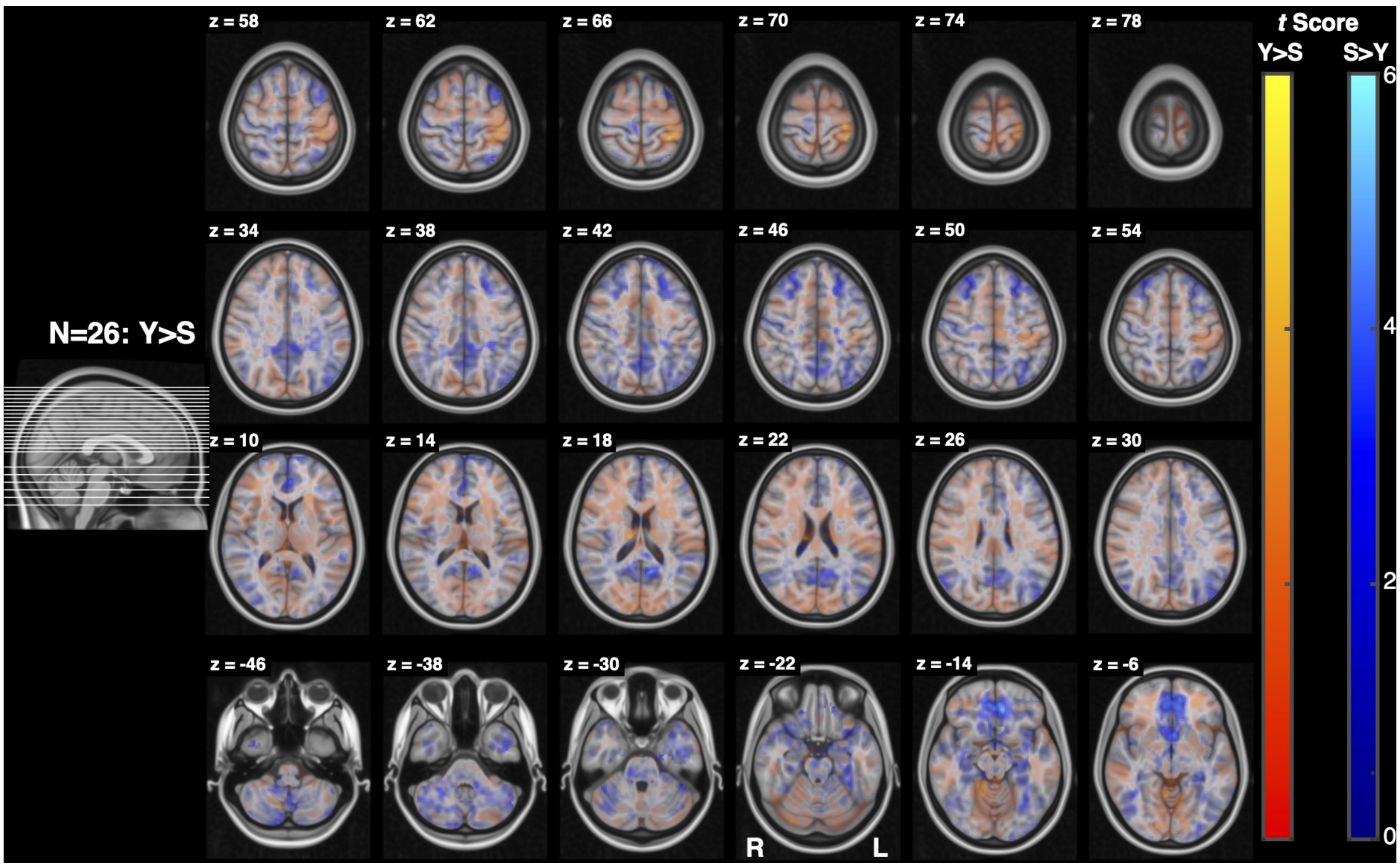

Figure 20: Cortical activation associated with the Yield>Slow contrast. A sagittal slice on the left shows the location of each axial slice of the brain shown on the right with the highest slice on the top right and the lowest slice on the bottom left. Functional activation is shown with lower t score voxels in red and higher t score voxels in yellow. Opacity is also modulated by t score and significant voxels are outlined in black.

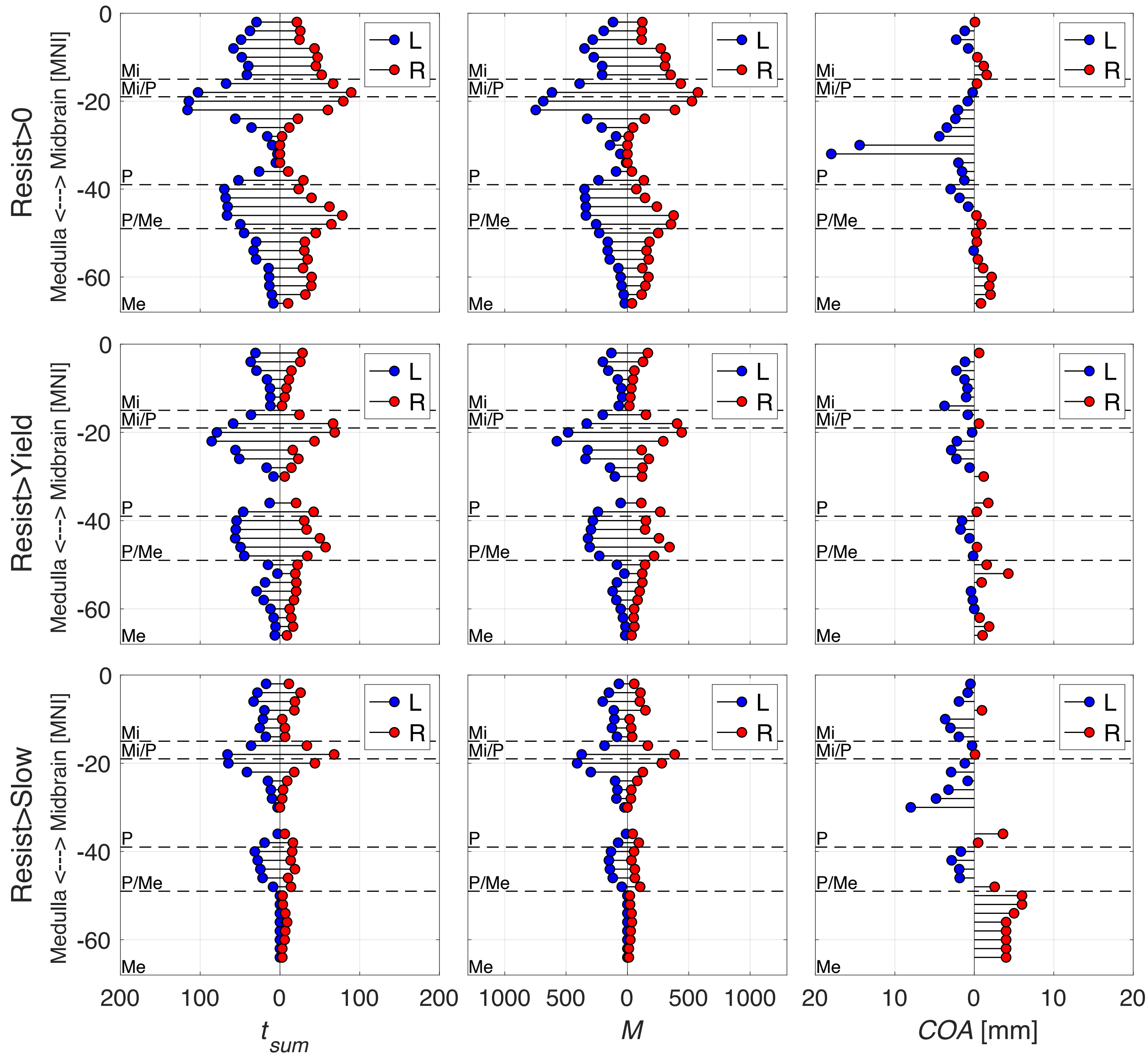

Figure 21: Laterality of significant (TFCE correction) activation in the brainstem. The three methods of quantifying laterality are shown with t_{sum} on the left, M in the middle, and COA on the right with the contrasts Resist on the top, Resist>Yield in the middle, and Resist>Slow on the bottom. Outcome values are on each x axis and axial slice location (z slice in MNI coordinates) are on each y axis. Horizontal dashed lines separate different sections of the brainstem by medulla, pons, midbrain, and their combinations.

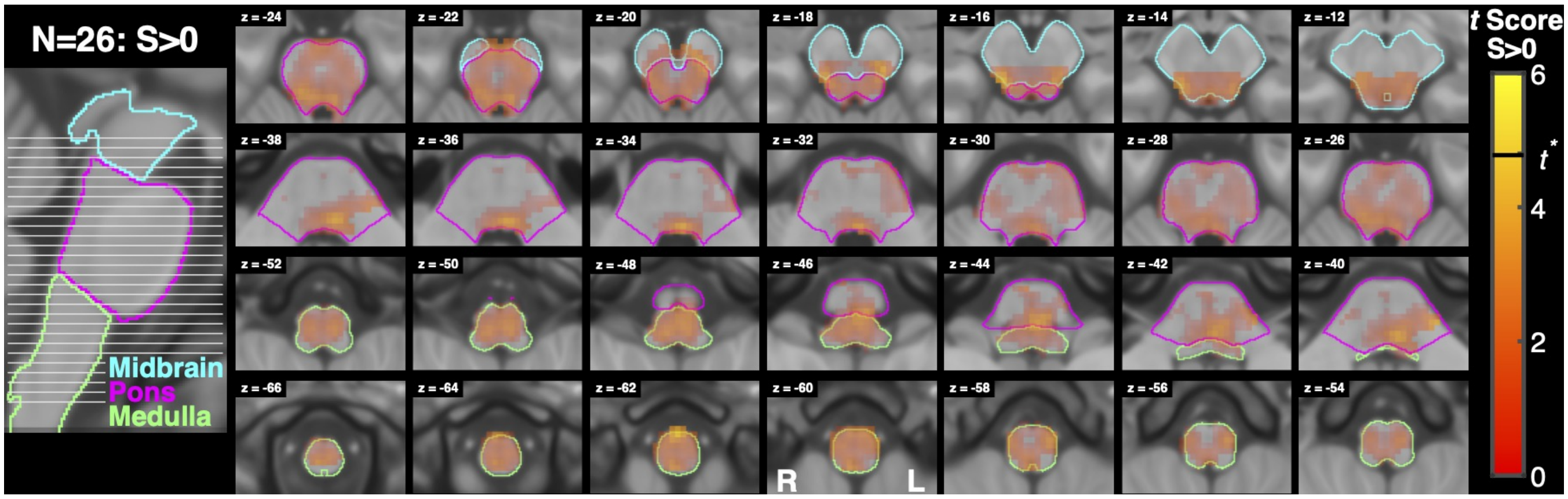

Figure 22: Activation associated with the Slow contrast using the more conventional general linear model without regressors for the rejected MEICA components and 4mm smoothing. A sagittal slice on the left shows the location of each axial slice of the brainstem shown on the right with the highest slice on the top right and the lowest slice on the bottom left. Three sections of the brainstem are outlined with different colors to facilitate orientation. Voxel color and opacity maps to t score (no lower limit), with significant voxels, thresholded using small volume correction, outlined in black. For this analysis $t^* = 4.8$.

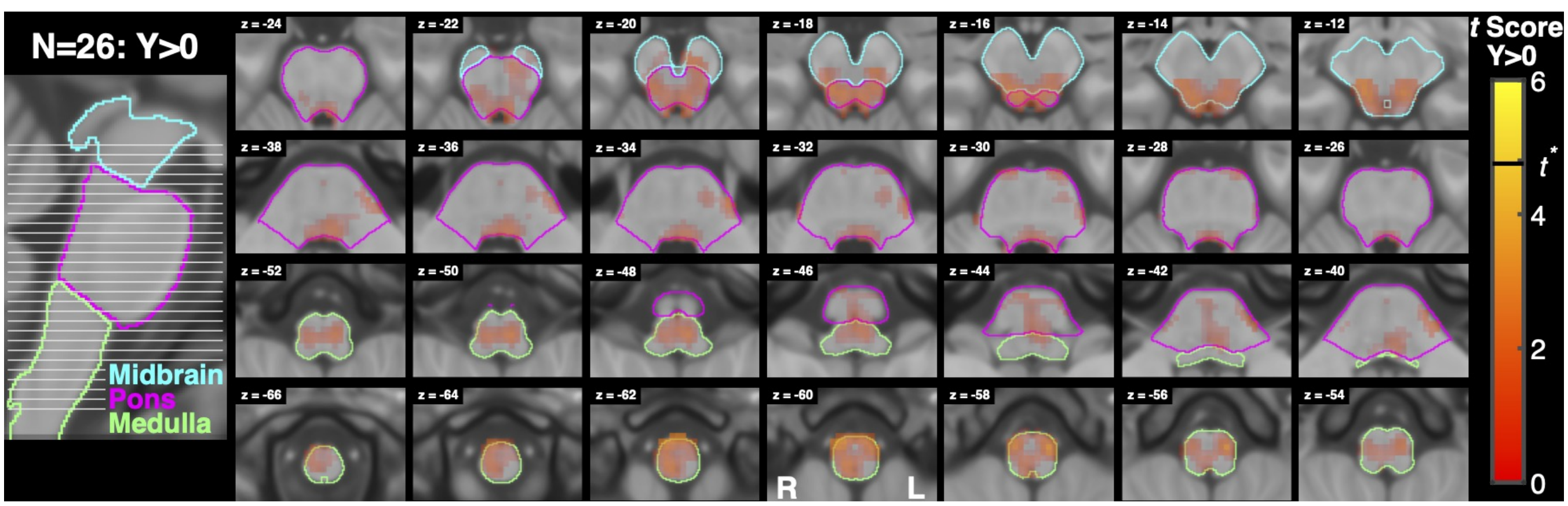

Figure 23: Activation associated with the Yield contrast using the more conventional general linear model without regressors for the rejected MEICA components and 4mm smoothing. A sagittal slice on the left shows the location of each axial slice of the brainstem shown on the right with the highest slice on the top right and the lowest slice on the bottom left. Three sections of the brainstem are outlined with different colors to facilitate orientation. Voxel color and opacity maps to t score (no lower limit), with significant voxels, thresholded using small volume correction, outlined in black. For this analysis $t^* = 4.8$.

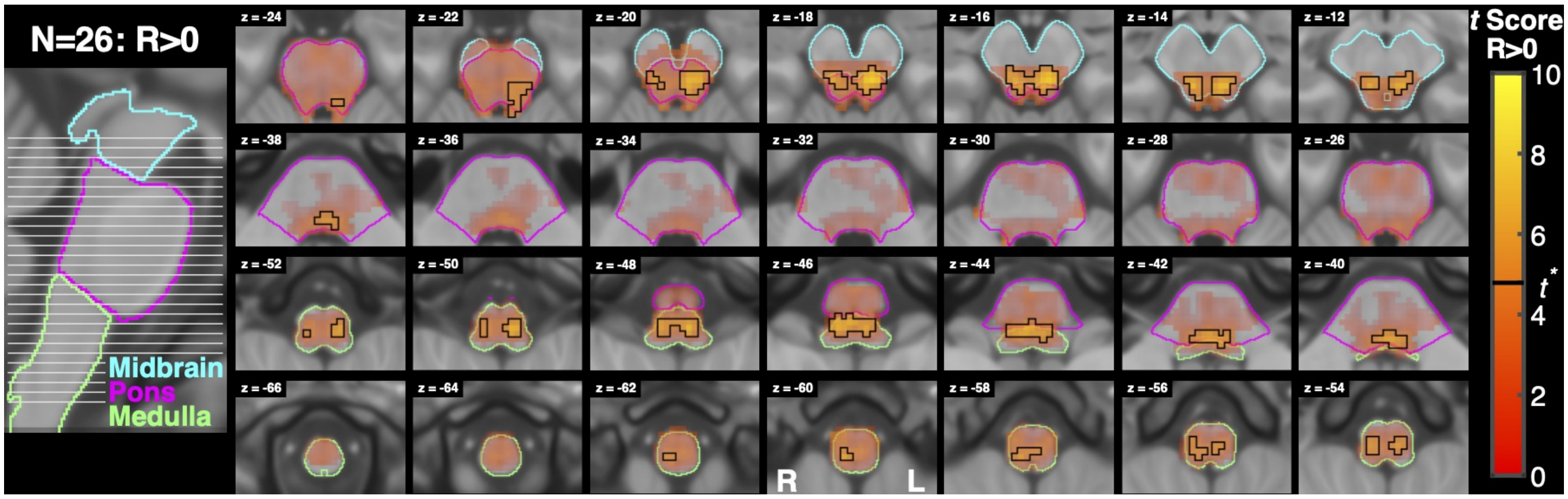

Figure 24: Activation associated with the Resist contrast using the more conventional general linear model without regressors for the rejected MEICA components and 4mm smoothing. A sagittal slice on the left shows the location of each axial slice of the brainstem shown on the right with the highest slice on the top right and the lowest slice on the bottom left. Three sections of the brainstem are outlined with different colors to facilitate orientation. Voxel color and opacity maps to t score (no lower limit), with significant voxels, thresholded using small volume correction, outlined in black. For this analysis $t^* = 4.8$.

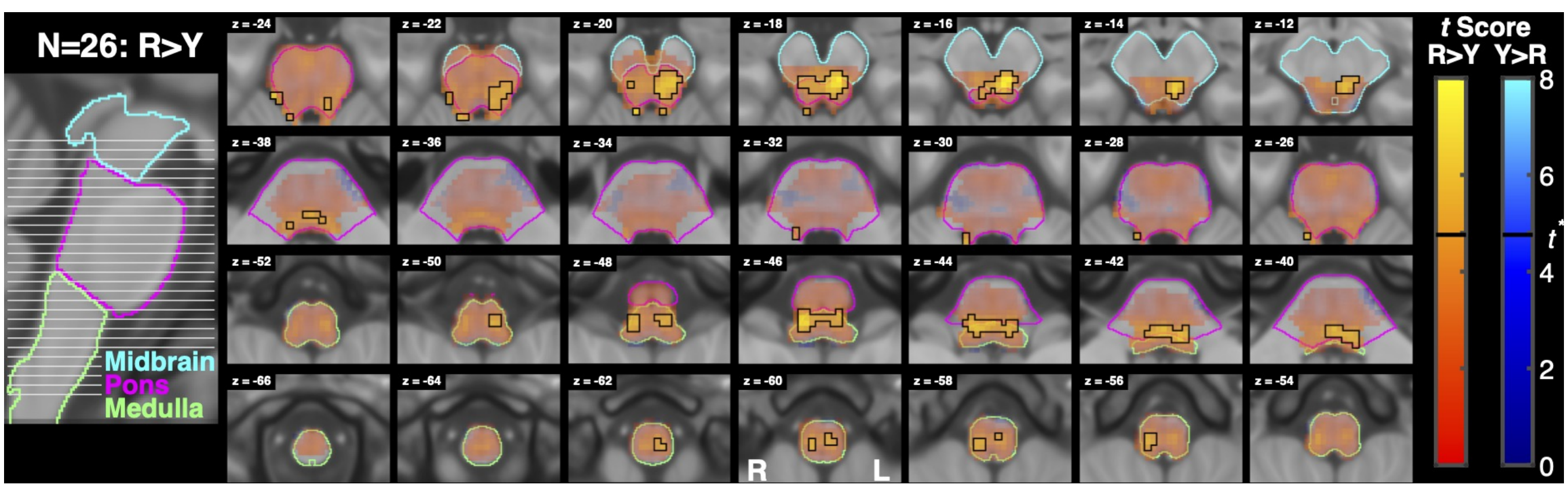

Figure 25: Activation associated with the Resist>Yield contrast using the more conventional general linear model without regressors for the rejected MEICA components and 4mm smoothing. A sagittal slice on the left shows the location of each axial slice of the brainstem shown on the right with the highest slice on the top right and the lowest slice on the bottom left. Three sections of the brainstem are outlined with different colors to facilitate orientation. Voxel color and opacity maps to t score (no lower limit), with significant voxels, thresholded using small volume correction, outlined in black. For this analysis $t^* = 4.8$.

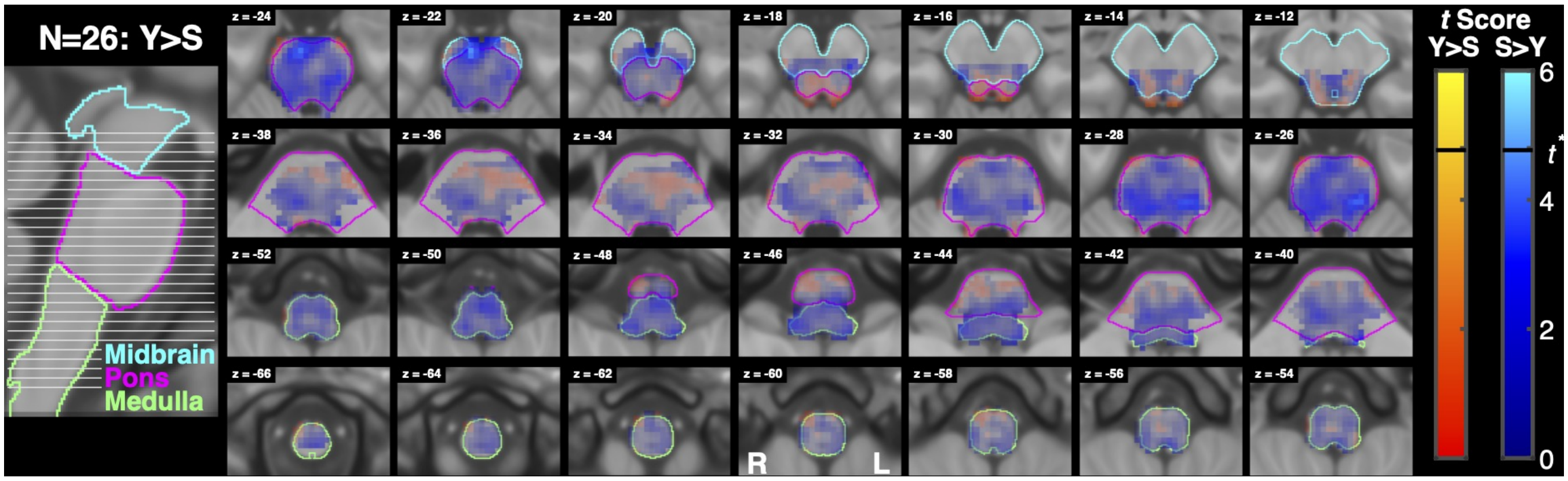

Figure 26: Activation associated with the Yield>Slow contrast using the more conventional general linear model without regressors for the rejected MEICA components and 4mm smoothing. A sagittal slice on the left shows the location of each axial slice of the brainstem shown on the right with the highest slice on the top right and the lowest slice on the bottom left. Three sections of the brainstem are outlined with different colors to facilitate orientation. Voxel color and opacity maps to t score (no lower limit), with significant voxels, thresholded using small volume correction, outlined in black. For this analysis $t^* = 4.8$.

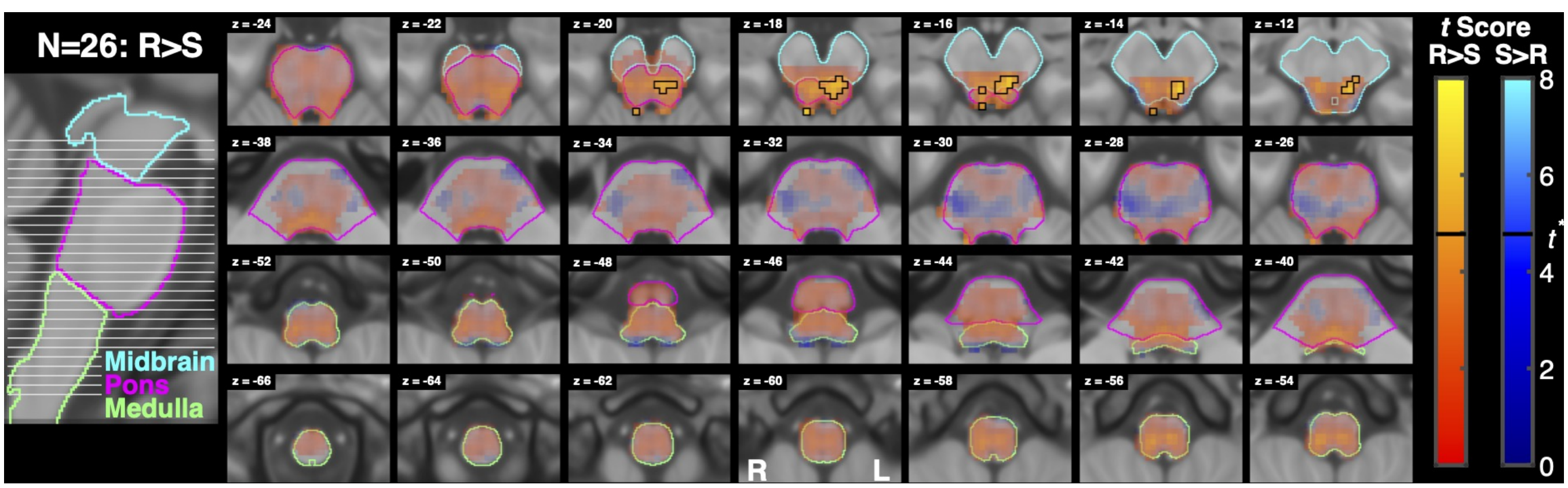

Figure 27: Activation associated with the Resist>Slow contrast using the more conventional general linear model without regressors for the rejected MEICA components and 4mm smoothing. A sagittal slice on the left shows the location of each axial slice of the brainstem shown on the right with the highest slice on the top right and the lowest slice on the bottom left. Three sections of the brainstem are outlined with different colors to facilitate orientation. Voxel color and opacity maps to t score (no lower limit), with significant voxels, thresholded using small volume correction, outlined in black. For this analysis $t^* = 4.8$.
